# Chemical Vapour Deposition of Graphene—Synthesis, Characterisation, and Applications: A Review

**DOI:** 10.3390/molecules25173856

**Published:** 2020-08-25

**Authors:** Maryam Saeed, Yousef Alshammari, Shereen A. Majeed, Eissa Al-Nasrallah

**Affiliations:** 1 Energy and Building Research Centre, Kuwait Institute for Scientific Research, P.O. Box 24885, Safat 13109, Kuwait; enasrallah@kisr.edu.kw; 2 Waikato Centre for Advanced Materials, School of Engineering, The University of Waikato, Hamilton 3240, New Zealand; y.namlan89@gmail.com; 3 Department of Chemistry, Kuwait University, P.O. Box 5969, Safat 13060, Kuwait; sh.sh.a.majeed@gmail.com

**Keywords:** graphene, deposition, CVD, growth, characterisation, flexible electronics, NEMS

## Abstract

Graphene as the 2D material with extraordinary properties has attracted the interest of research communities to master the synthesis of this remarkable material at a large scale without sacrificing the quality. Although Top-Down and Bottom-Up approaches produce graphene of different quality, chemical vapour deposition (CVD) stands as the most promising technique. This review details the leading CVD methods for graphene growth, including hot-wall, cold-wall and plasma-enhanced CVD. The role of process conditions and growth substrates on the nucleation and growth of graphene film are thoroughly discussed. The essential characterisation techniques in the study of CVD-grown graphene are reported, highlighting the characteristics of a sample which can be extracted from those techniques. This review also offers a brief overview of the applications to which CVD-grown graphene is well-suited, drawing particular attention to its potential in the sectors of energy and electronic devices.

## 1. Introduction

Graphene research is a relatively young field whose growth accelerated with the successful graphene exfoliation from bulk graphite by Professor Andre Geim and Professor Kostya Novoselov in 2004 at the University of Manchester [1]. Graphene, the basic building-block of graphitic materials, is a two-dimensional, single-atomic layer material consisting of six bonded sp^2^ carbon atoms that are tightly-packed in a honeycomb lattice with an interatomic distance of 1.42 Å [2]. Stacked graphene sheets form graphite (3D), a rolled sheet forms carbon nanotube (1D), and a spherical net forms a fullerene (0D). Among all sp^2^ carbon allotropes, graphene exhibits the most remarkable properties. It has a significant theoretical specific surface area (2630 m^2^/g) [3]. As suspended graphene, it has an ultrahigh electron mobility (~2 × 10^5^ cm^2^/Vs) [4]. It has high Young’s modulus (commonly reported value for defect-free single layer graphene is 1 TPa). Its thermal conductivity exceeds any other material with an exceptional value of 3500–5000 W/mK [5] and so does its electrical conductivity, with critical current density of 10^8^ A/cm^2^ [6]. Those extraordinary unique properties of graphene, including its conductivity, transmittance, flexibility and strength, enable its use in several applications such as electronics, composites, membranes and next-generation renewable energy technologies (e.g., solar cells). Subsequently, there have been numerous studies on the production of this remarkable material. However, one of the main challenges is the mass production of high-quality graphene with few or no contaminants or defects and large grain size at a reasonably low cost. Currently, various methods have been developed for graphene production, with each approach producing different dimensions, flake shapes, quality, and quantity. However, techniques that are suitable for production, in addition to their possible applications, are limited to the following: mechanical exfoliation (fundamental studies and research), liquid-phase exfoliation (mass production, low electrical quality), sublimation of silicon carbide (SiC) (high cost) and chemical vapour deposition (CVD).

Liquid-phase exfoliation relies on using energy to exfoliate bulk graphite within a solvent which has a suitable surface tension to stabilise the resulting graphene. The solvent used in the exfoliation process is typically non-aqueous, such as *N*-Methyl-2-pyrrolidone (NMP). However, aqueous solutions are also suitable if a surfactant is added. Originally, exfoliation energy comes via ultrasonic horn sonication; nevertheless, high shear forces are increasingly being used. The yield of these processes is typically a few percent; hence, centrifugation is used to obtain a significant fraction of monolayer and few-layer graphene flakes in the final suspension [7].

The controlled sublimation of SiC was an early method developed for graphene films in the electronics industry. Utilising this method, epitaxial graphene is achieved by thermal decomposition of a SiC substrate, using either e-beam or resistive heating. The process is conducted in an ultrahigh vacuum (UHV) to minimise contamination [8]. After the Si desorption has occurred, excess carbon on the surface of the SiC wafer rearranges to form a hexagonal lattice [9,10,11,12]. The main disadvantages of this method are its high cost, and it requires large amounts of Si for large-scale production.

Another method is CVD graphene using growth substrates and a hydrocarbon gas source. This is either through carbon diffusion and segregation in a metal substrate with high carbon solubility (e.g., Ni) or by surface adsorption in metal with low carbon solubility (e.g., Cu) [13,14]. However, among all the different graphene production methods, CVD graphene on metal substrates has shown the most promising results for producing monolayer graphene on large areas [14,15,16,17]. CVD is also a relatively inexpensive method, which produces a large area and high-quality graphene.

## 2. Chemical Vapour Deposition 

**Figure 1:**
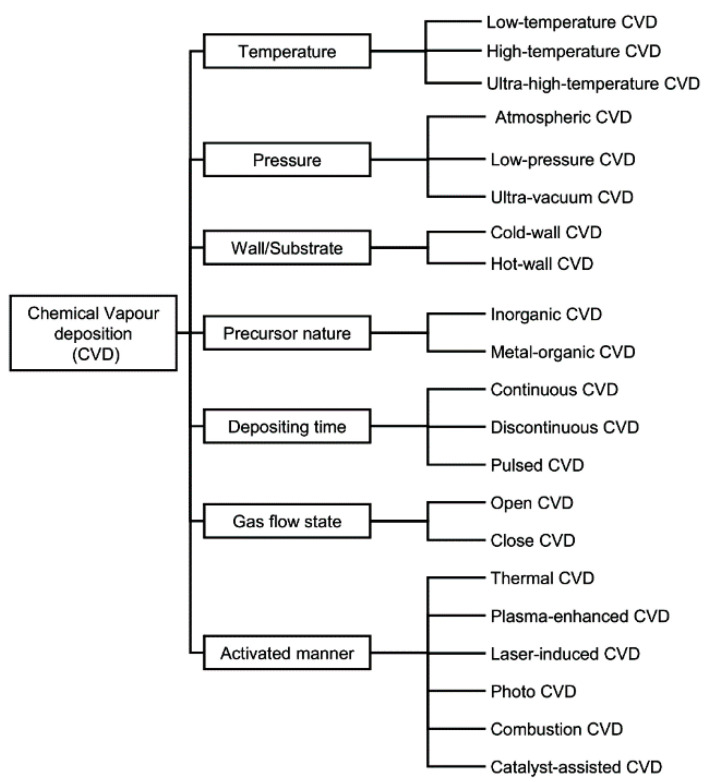
Classification of CVD methods (reprinted with permission from [23]).

CVD is the technique of depositing material as a thin film onto substrates from vapour species through chemical reactions. The process and types of the various possible chemical reactions that occur in a CVD reactor are governed by many complex factors, including the system setup, reactor configuration, gas feedstock, gas ratios, both reactor pressure and gas partial pressures, reaction temperature, growth time, temperature, etc. CVD is an extensively used bottom-up approach for the synthesis of few-layer and single-layer graphene films. Thermal CVD on metals was first reported in 1966 to grow highly crystalline graphite films on Ni substrates [18]. Later, deposition of a single layer of graphite was conducted using a CVD method on Pt surface by the hydrocarbon decomposition at 530 °C [19]. After the first isolation of graphene in 2004, the study of CVD’s potential for growing graphene films was successfully achieved by using LPCVD to grow graphene on Ir [20]. The reasons for the popularity of the CVD approach for graphene growth include ease of setup in research laboratories, successful long-term use in industrial settings, and the potential to scaleup fabrication [21]. Moreover, in consideration of both ecological and cost factors, the CVD approach is among the best available routes for the synthesis of graphene-based materials [22]. Today, a variety of different CVD methods are available that can be employed to synthesise graphene-based materials. According to the characteristics of the processing parameters (pressure, temperature, precursor nature, gas flow state, wall/substrate temperature, depositing time, and activation manner), these methods can be categorised into seven main types based on temperature, pressure, wall/substrate, nature of precursor, depositing time, gas flow state and activation/power source, as demonstrated in Figure 1 [23,24].

A range of precursors has been previously reported as suitable for the growth of graphene in the CVD reactor, for example, solid, liquid and gaseous carbon sources. Solid precursors may be directly loaded into the reactor chamber. Graphene film formation on a Cu surface was demonstrated by Sun et al. using spin-coated poly (methyl methacrylate) (PMMA) on Cu foil by thermal sublimation and the assistance of H_2_ and Ar [25]. A liquid carbon source was reported by Yao et al. using hexane [26]. In this method, the hexane was evaporated, and the vapour was introduced into the CVD reactor using bubblers. The vapour’s concentration is usually controlled by bubbling inert gas such as Ar through liquid hexane. However, both types of carbon sources can be challenging to control, so they were not used in this work. The most widely used carbon precursor is in gaseous form, such as methane (CH_4_) gas, which is introduced to the reaction chamber by a gas delivery system.

The CVD methods, as aforementioned, are classified based on the energy method used in the reactor to form the thin film deposition. The most used methods are (1) hot-wall CVD when the entire reaction chamber is heated by a furnace to provide the sufficient energy needed for annealing the growth substrate and the subsequent decomposition of the feedstock, (2) cold-wall-CVD where the growth substrate is directly heated to the desired growth temperature in an unheated reaction chamber, and (3) plasma-enhanced CVD (PECVD), where a high frequency voltage ignites plasma to low pressure gas (hydrocarbon feedstock) and the inelastic collisions in the reactor’s environment will lead to the formation of the reactive species needed for graphene deposition [21]. In this section, a review of those CVD methods for graphene growth will be discussed in terms of system setup, working mechanism, and the characteristics of the produced graphene.

### 2.1. Hot-Wall CVD

**Figure 2:**
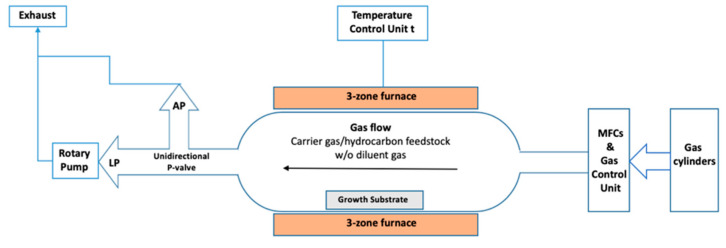
Schematic diagram of a typical hot-wall horizontal tube-furnace CVD system (LP: low pressure, AP: atmospheric pressure).

CVD is the technique of depositing material onto substrates from vapour species through chemical reactions. In CVD, the reactive gases are fed from cylinders into the reactor through a gas delivery system. The gas delivery system contains the valves, mass flow controllers (MFCs) and the gas-mixing unit, which mixes the different gases according to their required ratios before they enter the reactor. The reactor (reaction chamber) is where the chemical reaction takes place and where the products are deposited on substrates. Heating elements surround the reaction chamber, thus providing the necessary temperatures for the reaction to occur. The pressure is achieved as low or atmospheric pressure in the reactor by using either a pump for the evacuation of the reaction’s tube or a unidirectional relief valve to maintain atmospheric pressure inside the reactor. Finally, the by-products of the reaction and the non-reacted gases are removed by the gas delivery system through the exhaust (Figure 2) [27].

In the case of CVD graphene deposition, a metal substrate such as copper (Cu) is placed into a furnace tube made from quartz and heated under a flow of hydrogen at low vacuum or atmospheric pressure. The elevated temperature anneals the substrate which leads to enlargement of its grain size (typically in the range of 1–10 μm) [28], as well as reduction of the metal oxide film on the substrate surface [29]. During the growth stage, carbon is generally introduced by mixing a hydrocarbon gas such as methane (CH_4_) with hydrogen. The gases are then passed through the reactor at a predefined ratio. The partial pressures of the hydrocarbon and the hydrogen can be controlled by changing the reactor pressure or by adding an inert diluent gas such as argon (Ar) or nitrogen (N). After the reaction, the furnace is typically switched off and allowed to cool under a gas flow (e.g., H_2_, H_2_ + Ar) until the reactor reaches room temperature. This step is essential in order to avoid aggregation of the deposited carbon, which may result in multi-layer graphene or bulk graphite. It thus contributes to achieving a continuous graphene monolayer on the surface of the metal. 

**Figure 3:**
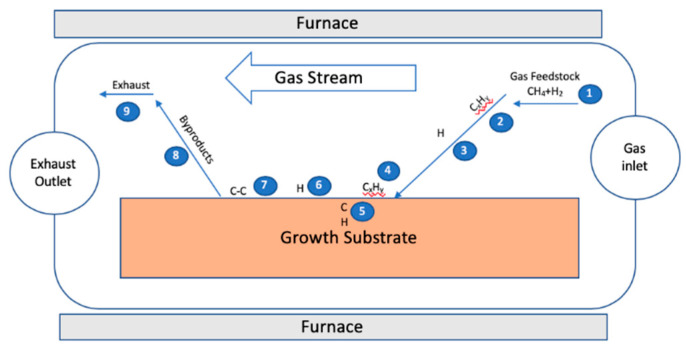
Schematic diagram of thermal CVD growth of graphene.

Figure 3 shows a schematic diagram of the CVD reaction for graphene from methane and hydrogen with each step numbered for clarification. The process starts with the transportation of reactants by convection in the gas flow (step 1) followed by their thermal activation (steps 2). Next, the reactants are transported by gas diffusion from the main gas stream through the stationary boundary layer (step 3). Then, the reactants are adsorbed on the surface of the substrate (step 4) and/or diffused into the bulk of the substrate (step 5), depending on the carbon’s solubility and the physical properties of the substrate used. In low carbon solubility substrates, the graphene growth step occurs on the surface where CH_4_ is catalytically decomposed and where the carbon atoms are adsorbed to form graphene film, while, in the high carbon solubility substrate, carbon atoms are diffused into the bulk, and the graphene growth step happens during the cooling step when carbon precipitates to the surface. Fast cooling is thus recommended to avoid the formation of multi-layer graphene. In surface processes (step 6), catalytic decomposition of reactive species occurs in addition to surface migration to attachment sites and other heterogeneous reactions. By-products are then desorbed from the substrate (step 7) after film growth. Finally, by-products are diffused through the boundary layer to the main gas stream (step 8) to be carried away by the force of convection to the exhaust system (step 9) [30].

#### 2.1.1. Role of Total Reaction Pressure

Hot-wall CVD can be mainly categorised into two types based on the total reaction pressure: low pressure CVD (LPCVD) and atmospheric pressure (APCVD). The LPCVD method was used for the growth of high quality carbon nanotubes (CNTs) in the late 20th century [31,32,33] and it has been used since then as an efficient approach for CNT synthesis [34,35,36,37]. Therefore, LPCVD was investigated as a promising method to grow graphene on transition metal substrates, and it was successfully reported to grow monolayer graphene on Cu foil (25 µm) by Li et al. [38]. Early studies of LPCVD of graphene on copper demonstrated that complete coverage of graphene can be achieved in less than three minutes, after which no further deposition is possible [39]. Later LPCVD studies indicated that the nucleation and growth of successive layers can proceed as long as a bare Cu surface is available, and that the new layers form underneath the existing graphene [40,41,42]. However, the use of CVD for graphene synthesis was pioneered by Reina et al. When the use of APCVD was reported even earlier than LPCVD, it demonstrated the ability to grow few-layer graphene on arbitrary substrates [43]. Conversely to LPCVD, it was reported that graphene grown using APCVD could lead to new layers being nucleated on top of the first deposited graphene layer [44]. APCVD tends to result in a few layers of graphene [26] or films with high defects density and regions of nonuniform thickness over the growth substrate that is usually in its solid state [45,46]. The tendency of producing few layers graphene in APCVD comes to the variations in the mass transport of carbon species in the gas stream inside the reactor across the extent of the substrate. While the improvement in graphene quality when the LPCVD system being used is due to kinetics at the growth substrate’s surface becomes more dominant than gas phase processes [45]. Each mechanism is governed by a barrier energy that needs to be overcome for a certain growth mechanism to proceed. Boundary layer, which is the region close to the surface of the substrate where the gas flow is stagnant due to laminar gas flow, and the thickness of this layer is important in the growth kinetics. Firstly, the hydrocarbon species are diffused through the boundary layer to reach the substrate’s surface. In the case of adsorption, the hydrocarbon species are adsorbed on the surface, followed by decomposition in order to provide the required carbon species to be diffused on the surface or in the bulk of the used substrate. The inactive hydrogen species are finally diffused as a by-product through the boundary layer back into the main gas flow. These steps are mainly classified into two scenarios or categories, the mass transport region where the diffusion through the boundary layer takes place and the surface reaction region [45]. However, boundary layers are known to be thicker in LPCVD than in APCVD. The diffusivity coefficient is larger in LPCVD than in APCVD. Low pressure leads to an increase in the diffusion coefficient, which causes a faster graphene growth rate. Nonetheless, the issue of depositing few layers graphene in APCVD can be improved by highly diluting carbon in hydrogen [45,47,48] or increasing the growth temperature above the melting point of the growth substrate [49,50]. While the surface diffusion of reaction limits the graphene growth to a single layer upon coverage of the copper surface, it has been shown that more layers can be grown on copper [51,52]. Cho et al. managed to grow bilayer and multilayer graphene by controlling the growth pressure during the CVD process [51]. Studying several growth pressures showed that single layer graphene growth is dominant at low pressures. However, upon reaching pressures more than 50 mbar, bilayer and monolayer graphene become dominant.

Another interesting observation when using low or atmospheric pressure CVD systems is the stacking in a few layers of graphene, it shows that LPCVD leads to turbostratically stacking while APCVD to Bernal stacking [53]. Furthermore, as aforementioned that kinetics on the substrate’s surface dominated the graphene formation in LPCVD, it has been shown that when the CVD process is performed at low pressure, the surface morphology of the substrate plays a crucial role in determining the growth rate of graphene, as in the case of using Cu substrate, its sublimation at low pressure and high temperature affects the desorption rate and nucleation rate [54] (growth substrate will be discussed thoroughly in Section 3). 

#### 2.1.2. Role of Hydrogen and Hydrocarbon

Hydrogen is widely used in the annealing step since it removes the oxide layer on the metal surface. Therefore, it plays an essential role in the cleaning and crystallisation of metallic substrates. Molecular hydrogen (H_2_) plays a crucial role in the graphene growth step. When the metal surface is annealed under H_2_ flow, dissociative chemisorption of H_2_ and/or H takes place in the substrate that later competes with CH_4_ for chemisorption. Atomic H creates sites that lead to the dehydrogenation of CH_4_ and carbon radicals on the substrate’s surface. This formation of active surface-bound C_y_H_x_ results in graphene growth [30,48,55]. Consequently, hydrogen slows down the deposition kinetics of graphene on Cu surfaces by blocking surface sites and reduce their number for CH_4_ chemisorption; however, it enhances the provision of CH radicals needed for graphene nucleation.

The annealing step under the flow of hydrogen and this competitive dissociative chemisorption role shows differences in behaviour between Ni and Cu surfaces. The hydrogen interaction with the two substrates affects the CH_4_ chemisorption kinetics as they have different levels of hydrogen solubility [48]. This proves that hydrogen diffusivity and solubility in the substrate determines its effectiveness in growth kinetics. The observation of Losurdo et al. showed that hydrogen diffuses in the Cu substrate while it recombines on the Ni surface in agreement with the difference in their H_2_ diffusion coefficients [48]. In addition, the higher hydrogen flow rate was shown to decrease the growth rate of graphene since the high H_2_ feed in the reactor leads to a reduction of active sites on the surface of the Cu substrate. In Ni, the opposite H_2_ effect was observed, as at the growth temperature, active sites on the surface of the Ni substrate increased with the increase of the H_2_ flow rate. This is a reason for the migration of the subsurface H to the surface leading to H-H recombination and desorption from the Ni surface, thus leaving more available active sites for CH_x_ dissociative chemisorption [48,56]. Interestingly, the purity of the used hydrogen was demonstrated to play a crucial role in graphene etching after the growth step. A study by Choubak et al. showed that the etching effect of hydrogen disappears when purified ultra high purity (UHP) grade hydrogen is used [57]. On the other hand, graphene etching due hydrogen flow in cooling steps was observed when unpurified UHP hydrogen was used. This suggests that oxidising impurities are responsible for etching reactions. Thus, the amount of oxidising impurities should be carefully considered to achieve a balanced growth and etching rates. Therefore, the catalytic substrate has a direct effect on the structure of graphene, its growth behaviour, crystallinity, layer number, size distribution, and nucleation density [58] in addition to its role in etching as well due to the presence of oxidising impurities. Hence, the substrate pre-cleaning process decreases the concentration of impurities and provides a cleaner and more homogenous growth substrate [29]. In a recent study by Ibrahim et al., different hydrogen flow rates were used in the annealing step showing that the surface morphology of the growth substrate (Cu in this case) in the absence of hydrogen exhibited step-like structure while, in the presence of the hydrogen, the surface becomes smoother with some surface defects [59]. Those observations profoundly influenced the morphology of the grown graphene, as the no-H_2_ annealing resulted in a wide-spaced steps graphene structure while the H_2_ annealing led to narrow-spaced wrinkles structure, which is more favourable. However, annealing under hydrogen flow created surface defects promoting the deposition of multilayer graphene. Thus, a lower concentration of hydrogen led to a smoother surface and limited the deposition to mono-bilayer graphene [59].

**Figure 4:**
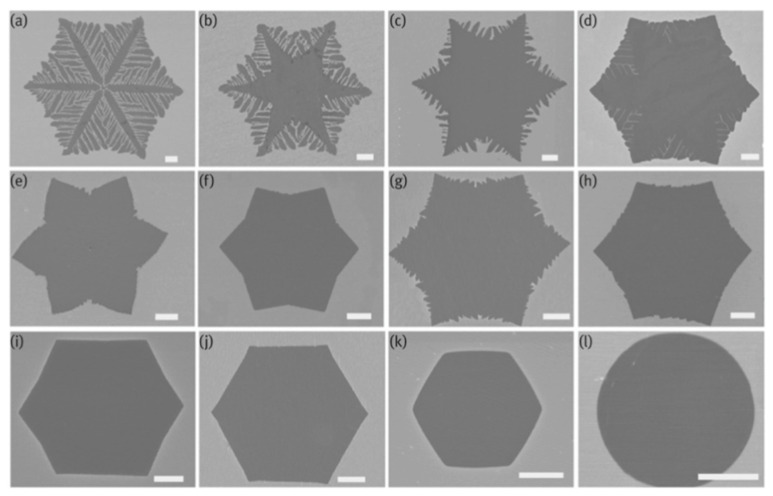
SEM images illustrate the evolution of the shape of graphene domains using different H_2_/CH_4_ ratios. (**a**–**h**), H_2_/CH_4_ ratios are 20, 30, 40, 60, 70, 80, 100 and 120, respectively, while the CH_4_ flow rate is kept constant at 0.5 sccm. The flow rates of H_2_/CH_4_ for (**i**–**l**) are 200/2, 200/4, 300/5 and 300/22 sccm, respectively. All scale bars are 5 µm (reprinted with permission from [63]).

Moreover, hydrogen plays a role in limiting the graphene thickness by carbon etching when removing the weak C-C bonds [60,61]. It was also found that, at a high H_2_ flow rate, a selective etching occurs to trim the graphene edges during its growth process [62]. Another report on the effect of hydrogen and hydrocarbon flow rates on the morphology of the grown graphene domains was conducted by Wu et al. showing that the shape of the graphene flakes grown on liquid Cu can be precisely tailored by tailoring the diluent gas/H_2_ gas flow rate [63]. They have used the APCVD method to grow graphene on liquid Cu using CH_4_ as the hydrocarbon feedstock and a mixture of Ar and H_2_. Typically, graphene domains with dendritic shape result in a low H_2_/CH_4_ ratio due to diffusion-limited growth, while increasing the H_2_/CH_4_ ratios tend to lead to the formation of compact hexagonal graphene domains due to the edge attachment limited growth until eventually reaching regular hexagonal graphene domains. Furthermore, the edges of the hexagonal graphene flakes had a positive curvature when higher CH_4_ flow rate was used until it reached the formation of circular graphene flakes (Figure 4). 

Hussain et al. studied the effect of hydrogen in LPCVD on graphene growth [64]. Their results determined the effect of hydrogen on the physical and electrical properties of the grown graphene. Higher hydrogen flow rates led to an increase in the grain growth, while graphene using lower hydrogen flow rates exhibited higher surface contamination due to more oxygen-related functional groups (e.g., amorphous and oxides carbon) [64]. 

**Figure 5:**
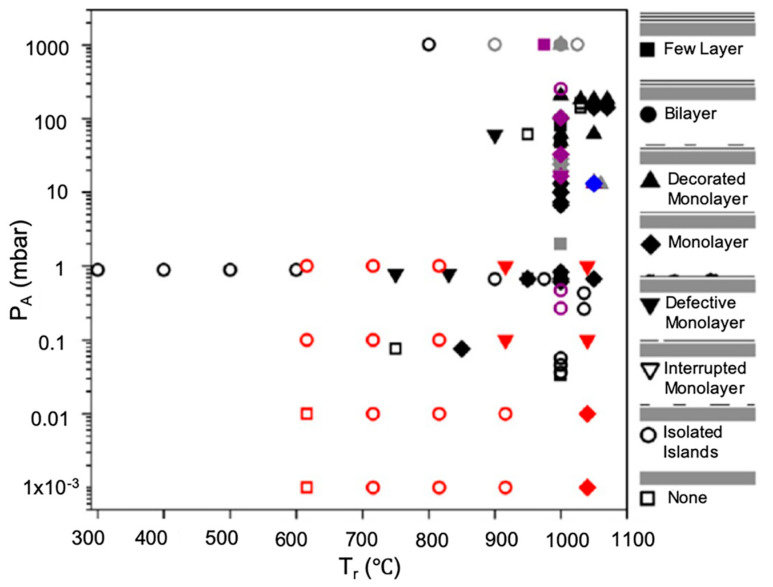
Coverage type for CVD of graphene versus growth temperature and pressure of active species as reported in the literature and Lewis et al. study. The data points span a range of different R_CH_ denoted by the color: black: 1 > R_CH_ > 0.1; gray for 0.1 > R_CH_ > 0.01; purple for 0.01 > R_CH_ > 0.001 and blue for 0.001 > R_CH_ > 0.0001. The red data points correspond to the work done by Lewis et al., where R_CH_ = 0.2 (reprinted with permission from [65]).

The hydrocarbon feedstock (e.g., methane) is an essential parameter in the CVD growth process of graphene. Methane (CH_4_) concentration described in terms of flow rate or partial pressure directly affects the growth kinetics and thermodynamics determining nucleation density and the graphene domain size. Early reports highlighted the correlation between CH_4_ concentration and the nature of the produced graphene when using LPCVD and APCVD systems. Bhaviripudi et al. investigated the APCVD graphene growth on Cu substrate using a range of CH_4_ flow rate. Their results demonstrated that monolayer graphene was obtained when using low methane concentration, but at higher CH_4_ concentration, multilayer graphene was observed to be deposited on top of monolayer graphene [45]. It was also observed that the grown graphene tends to have a higher number of defects at higher CH_4_ concentration in APCVD. This is explained by the likely increase of particulate concentration from gas reactions when a higher methane flow rate is used. Lewis et al. studied the influence of gas-phase equilibria on the grown graphene via hot-wall-CVD [65]. The partial pressure of active species (partial pressure of hydrogen and hydrocarbon P_A_) and C:H ratio (R_CH_) were investigated in various studies, and it was concluded that monolayer graphene is favourable when R_CH_ is within the range 4 × 10^−4^–0.25. The space of parameters was demonstrated by Lewis et al. summarising the type of graphene coverage (in terms of produced number of layers) using different P_A_ and R_CH_ for different growth temperatures (Figure 5). When diluent gas was used to lower the partial pressure of the active species in APCVD, all reported data points had R_CH_ < 0.02. The contrast with LPCVD is dramatic; most LPCVD experiments use a P_A_ within the range 0.1–1 mbar with R_CH_ > 0.1. 

**Figure 6:**
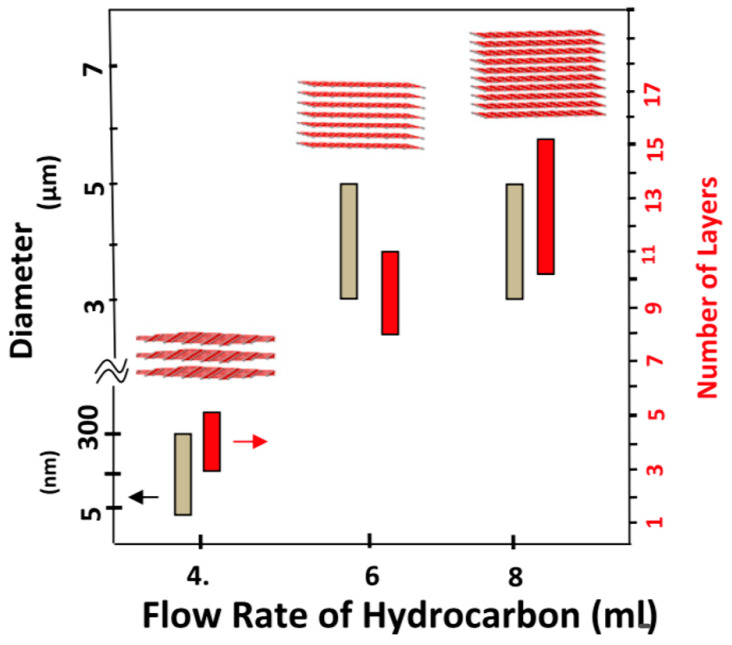
Flow rate of acetylene as the hydrocarbon feedstock versus the diameter and the number of layers produced in CVD has grown graphene (reprinted with permission from [66]).

Acetylene was also used as a choice of a hydrocarbon feedstock to grow graphene via CVD [66,67,68,69]. Acetylene has a higher pyrolysis rate compared to methane; thus, it may reduce the defect density in the graphene grown on metals due to the vacancy defects healing phenomena [70]. Wang et al. reported that CVD graphene grown using acetylene exhibits lower defect density when C_2_H_2_ flow rate is decreased [69]. In another study, Dervishi et al. investigated the role of hydrocarbon concentration on CVD grown graphene using acetylene as the hydrocarbon feedstock [66]. They demonstrated that controlling the hydrocarbon flow rate allows one to tailor the number of layers and the diameter of domains (Figure 6).

The investigation of alternative precursors to widen the choice window for CVD graphene growth included liquid precursors such as methanol, propanol and ethanol vapour, showing that ethanol is the most sensible choice [71,72,73,74]. Ethanol decomposition occurs at a lower temperature than methane [74] which contributes to reducing graphene growth temperature. An excellent review by Faggio et al. discusses the recent advancements on the growth of CVD graphene from ethanol vapour [57]. They showed that graphene quality is controllable by tuning growth conditions using ethanol vapour, which consequently tune the electrical and optical graphene characteristics.

#### 2.1.3. Role of Growth Temperature 

The CVD growth temperature is one of the most influential factors on the physicochemical mechanisms of graphene nucleation and growth, including the atomic level steps. In other words, the process of graphene nucleation and the proceeding growth is a result of adsorption, desorption and adsorbate (active atoms) migration mechanisms; each of these is highly influenced by the process temperature. Reports that discuss surface kinetics in greater detail showed that increasing the reaction temperature accelerates the growth rate of the CVD graphene film [40,75]. In principle, it is believed that graphene nucleation density decreases at higher temperatures [40,76,77]. Lui et al. suggested that increasing the CVD process temperature will lead to a decrease in the substrate’s surface roughness, which reduces the active sites for nucleation and improves the mobility of the active species [76]. CVD reaction temperatures close to the melting point of Cu or above (T_m_ of Cu = 1084 °C) tend to result in high-quality graphene with monolayer, uniform, crystalline, continuous and low defects density within the graphene film, which can be due to the rapid dehydrogenation rate of the hydrocarbon feedstock and/or to the improved probability that active carbon species have on the sufficient energy needed to surmount the energy barrier and attach to the surface for the growth of the graphene film [49,50,59,75].

**Figure 7:**
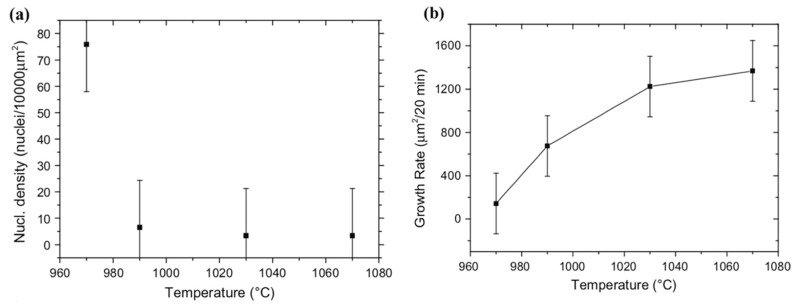
Analysis plots showing (**a**) nucleation density versus growth temperature, (**b**) nucleus growth rate versus growth temperature (reprinted with permission from [78]).

Chaitoglou and Bertran investigated the effect of the temperature on CVD graphene growth using a temperature range of 970–1070 °C; the temperature choice was made as lower temperatures led to no graphene nucleation while the higher limit was close to the Cu melting point [78]. They studied the temperature effect on the process kinetics of the graphene growth based on the SEM images by analysing the morphological features considering the nucleation density, growth rate and graphene surface coverage. Their results showed that the highest nucleation density occurs at the lowest growth temperature (75 nuclie/10,000 µm^2^ at 970 °C) while the lowest nucleation density occurs at the highest temperature (3.4 nuclei/10,000 µm^2^ at 1070 °C) (Figure 7a). At higher temperatures, the graphene growth rate rapidly increased (144 µm^2^/20 min at 970 °C compared to 1369 µm^2^/20 min at 1070 °C) (Figure 7b).

**Figure 8:**
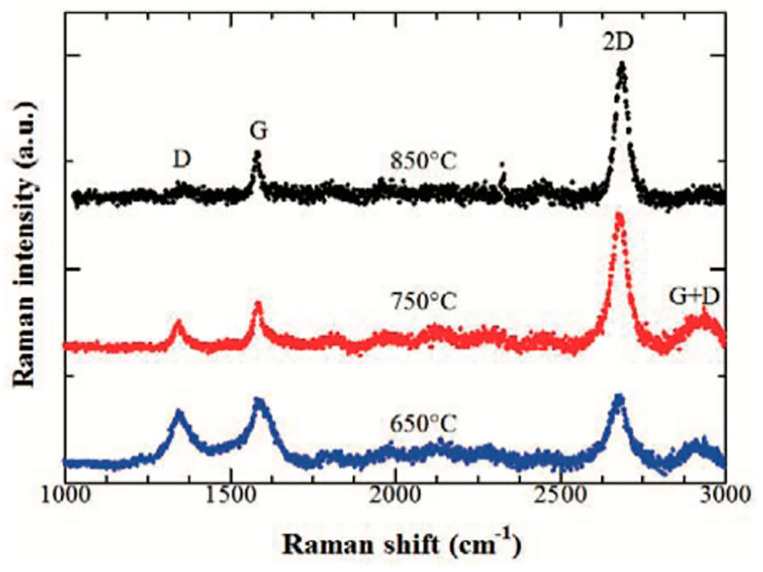
Raman spectra of the CVD grown graphene samples as a function of temperature (reprinted with permission from [79]).

Dapthon and Chaisitsak studied the effect of growth temperature on CVD graphene growth, using a range of 650–950 °C [79]. The graphene quality was characterised by the obtained Raman spectra and analysing the peaks’ intensity ratios of I_2D_/I_G_ and I_D_/I_G._ The highest graphene quality was obtained at a growth temperature of 850 °C with I_2D_/I_G_ of 7.45 and I_D_/I_G_ of 0.35 and narrow full-width-half-maximum (FWHM) of Lorentzian-shaped 2D peak of 38.88 cm^−1^ which are consistent with high-quality monolayer graphene with low defects density. Conversely, the lowest growth temperature resulted in a thick and highly defected graphitic film with I_2D_/I_G_ of 0.68 and I_D_/I_G_ of 0.76. Furthermore, the graphene grain size drastically decreased at lower growth temperature (L_a_ = 22.18 nm at 650 °C) compared to the ones obtained at a higher temperature (L_a_ = 47.51 nm at 850 °C), this is shown in Figure 8.

In agreement with the aforementioned results, Alnuaimi et al. studied the effect of graphene growth temperature using a cold-wall CVD reactor, showing that multilayer nucleation density is reduced at higher process temperature [80]. The investigated growth temperature varied from 1000 to 1060 °C; multilayer graphene regions were grown at 1000 °C while the nucleation density was reduced by more than 50% at 1060 °C where higher defects density was observed at lower growth temperature.

### 2.2. Cold-Wall CVD

Many experts consider the cold-wall CVD method to be a promising approach for large scale production of graphene materials with reduced production cost and very high throughput. In this technology, the entire reaction chamber is not heated, but only the substrate and the chamber walls remain at room temperature. There are several available options for heating the substrate, including passing a current through the substrate itself, induction heating, and using a heater adjacent to the substrate. The cold wall CVD method has some critical advantages over the hot wall approach, namely a less sophisticated design of the reactor, a relatively short deposition time of only a few minutes, a rapid sample heating time, and a rapid sample cooling time [24,81,82]. Faster heating and cooling are beneficial for achieving fast growth of graphene, which means significantly reduced costs associated with maintaining process conditions (pressure, temperature and hydrogen flow) [83].

Moreover, the reduced gas-phase chemical reaction due to lower reaction temperatures results in no particulate contamination, thereby enabling better control of the quality of graphene films [30]. Only requiring local heating, the cold wall CVD reactor has a much lower heat capacity compared to the hot wall system and, as such, consumes less power [81,84]. According to estimates by Bointon et al., the cost reduction using the cold wall CVD method for the synthesis of graphene performed in the ultra-high vacuum regime can reach up to 99% [81].

**Figure 9:**
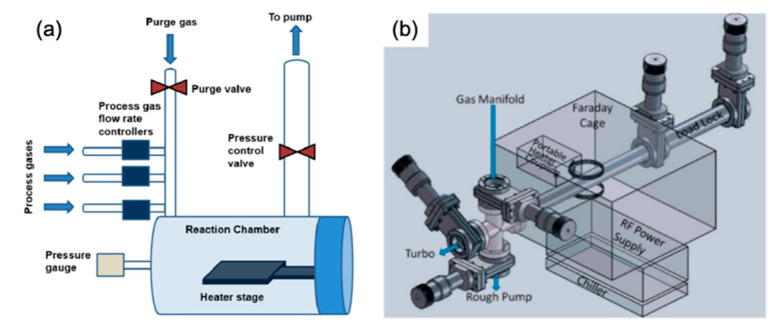
(**a**) a scheme of the cold wall CVD system used for graphene growth [81] and (**b**) the cold wall CVD system based on radio frequency magnetic inductive heating (reprinted with permission from [85]).

The literature describes several different types of cold wall CVD systems that have been developed for the synthesis of graphene, including the rapid thermal annealing CVD process using a halogen lamp, resistively heated stage CVD, magnetic induction heating CVD and Joule heating CVD [80,81,85]. Figure 9a,b illustrates a schematic representation of the cold wall CVD system and the apparatus diagram for a cold wall CVD reactor based on radio frequency induction heating, respectively.

**Figure 10:**
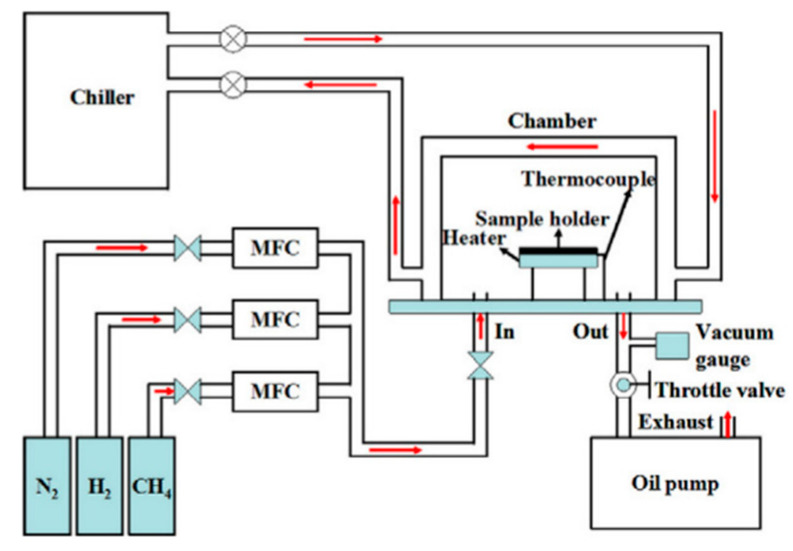
Diagram of the resistively heated stage cold wall CVD system (reprinted with permission from [88]).

A significant part of publications on the cold wall CVD process is devoted to the effect of various process parameters on the growth of graphene. Sarno et al. successfully obtained a graphene film on a Cu foil using the cold wall CVD method, which was then transferred to a SiO_2_/Si and polyethylene terephthalate (PET) substrates [83]. In their attempts to obtain ordered graphene, the authors investigated the effect of various modifications to the operating conditions, including the following: increased synthesis time (for the more complete the graphene coverage); increased pretreatment time (to facilitate the formation of Cu grains of needed size); optimisation of the low-pressure vacuum conditions (to control chemical reaction, promote stream diffusion and decrease material flow); and altering the gas flow rates, such as the concentration of diluent gas as they reduced the argon flow rate (to increase the C-conversion rate and reduce partial pressure of CH_4_). Similar to the effect of growth substrate in hot-wall CVD, the increase of the Cu grain size and the uniform crystallographic orientation of the surface were established to be key factors affecting the growth of graphene films by cold wall CVD and their quality. Furthermore, according to the author’s estimations, a prominent advantage of the proposed cold wall CVD technique is the significantly reduced energy costs due to much shorter synthesis times (6−12 min) and low vacuum conditions (1.1 × 10^−2^ bar) [83]. Chang et al. demonstrated the feasibility of a resistively heated stage (pyrolytic graphite/pyrolytic boron nitride) cold wall CVD for the synthesis of single-layer and few-layer graphene sheets on Ar plasma treated Cu and Ni foils using CH_4_ as a gaseous precursor. The work concludes that, although graphene films deposited on Ni foil are similar to films deposited on a Cu substrate, they are characterised by a higher number of defects [86]. Huang et al. successfully prepared high-quality graphene films in the cold wall CVD system with a rapid thermal processing heater using the Ni foil as the metal substrate, which was heated in the pyrolytic graphite/pyrolytic boron nitride heater up to a temperature of 1000 °C at a heating rate of 100 °C/min for 30 min in the gaseous atmosphere consisting of N_2_ and H_2_ (schematic diagram of the used system is illustrated in Figure 10). The article concludes that the growth time directly affects the quality of graphene films and that their sheet resistance and optical transmittance increase significantly with the decreasing concentration of H_2_ [87].

Machac et al. successfully employed the cold wall CVD reactor to prepare graphene films with unusual structure (between bilayer and single-layer graphene) using the Cu foil as the metal substrate and CH_4_ as a source of carbon. The graphene films showed a large resistivity measured by the Van der Pauw method. The graphene material transferred to a dielectric substrate (SiO_2_) displayed a well-resolved band structure, which evidenced that the obtained material was undoped [82]. A study by Dong et al. focuses on the in situ growth of patterned graphene structures via a vertical cold wall CVD reactor using a SiO_2_/Si substrate and Ni-Cu alloy sacrificial layer as a metal catalyst at 800 °C. The Ni-Cu alloy is particularly suitable to be used as a growth substrate for graphene at low temperatures due to its catalytic nature, low carbon solubility (about 4.3 times lower than that of pure Ni), and more uniform grain size compared with Cu and Ni. Due to these advantages of the Ni-Cu alloy, the graphene film obtained by the proposed method exhibited excellent uniformity and a high monolayer ratio [89].

Das and Drucker reported the possibility of tailoring the average size of graphene crystallites and the nucleation density of graphene grown using cold wall CVD by varying growth parameters. Experiments on the cold wall CVD synthesis of graphene using Cu films electrodeposited on W substrates revealed that the mechanisms of nucleation and growth of graphene are similar to those observed in the hot wall CVD method [89]. The influence of such process parameters as hydrogen partial pressure and the H_2_/CH_4_ ratio on the growth of bilayer graphene in low-pressure cold wall CVD reactor was investigated in a study by Mu et al. [84]. The authors found that the effect of hydrogen partial pressure was opposite to its effect in hot-wall CVD. High partial pressure of H_2_ was found to suppress the nucleation during the initial stage of the CVD process and lead to a decreased growth rate of both bilayer and single-layer graphene. Another finding is that the H_2_/CH_4_ ratio has a direct influence on the atomic structure of the bilayer graphene [84]. As noted by Liu and Fu, the use of liquid substrates in the cold wall CVD process makes it possible to achieve a quasi-atomically smooth surface, which, in turn, enables the growth of uniform layers of graphene. A characteristic feature of this approach is unique etching and growth kinetics due to the very fast surface diffusion rates, allowing the development of graphene grains with novel morphologies [90].

The cold wall type reactor can be applied not only for conventional CVD processes but also for plasma-enhanced CVD (PECVD). A study by Kim et al. explores a cold wall, microwave plasma-assisted CVD system for the synthesis of graphene films on polycrystalline Ni foil. A total pressure of 20 Torr, a range of substrate temperatures (from 450 to 750 °C) and various mixing ratios of CH_4_ and H_2_ were chosen as the operating parameters of the CVD process. The authors concluded that higher temperatures of the substrates lead to the growth of graphene sheets of higher quality [91]. 

Recently, another interesting study was conducted by Chen et al. for the first time to encapsulate Cu particles by few layer graphene grown via cold wall CVD [92]. The synthesis of graphene-encapsulated Cu nanoparticles (GCPs) in their work was inspired by the core–shell structure in order to replace Ag as a functional filler in polymer composites. The obtained GCPs exhibited high thermal stability (~179 °C), while the excellent thermal conductivity of graphene as well as its oxidation resistance nature enabled its use in thermally conductive adhesive (TCA). The GCPs in TCA showed a remarkable enhancement in performance by 74.6% compared to Ag, which is the conventionally used functional filler in TCA.

### 2.3. Plasma-Enhanced CVD

**Figure 11:**
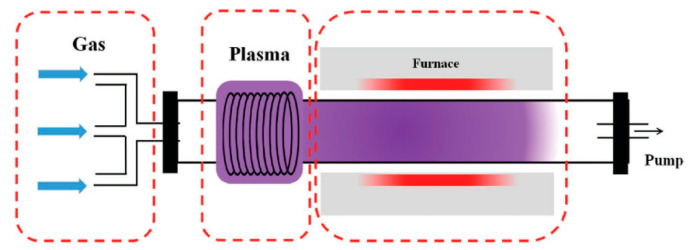
A schematic of a general PECVD setup (reprinted with permission from [95]).

Plasma-enhanced CVD (PECVD) is a method for graphene growth with the ability to grow graphene at a lower temperature with better control in the nanostructure, faster growth and higher selectivity producing more ordered/patterned materials [93]. In this technique, the plasma, the fourth state of matter, can generate species which are more reactive than ground-state atoms or molecules such as excited molecules and ions, photons, free radicals and energetic electrons at low or atmospheric pressure [94] that can dissociate the gas carbon source at relatively low temperatures in addition to lowering the energy barriers for nucleation and growth process in CVD. Moreover, PECVD induces the graphene growth on dielectric substrates rather than on the surface of the catalytical metal substrate, protecting the graphene from contamination and structural defects which might occur in the graphene transfer process and hence reduce its performance in electronic applications. [95]. There are three main parts in the experimental setup of PECVD, including gas, plasma generator and vacuum heating chamber. The general setup for PECVD is presented in Figure 11 [95].

Plasma generators can be classified into three types; microwave (MW) (~2.45 GHz), radio frequency (RF) (~13.56 MHz) and direct current (DC). Here, we review recent advances in the synthesis of graphene by PECVD techniques. 

#### 2.3.1. Microwave Plasma

**Figure 12:**
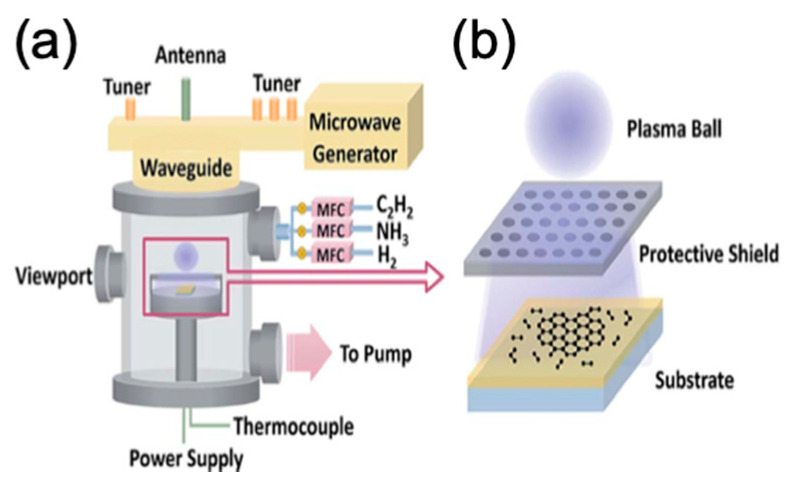
A schematic of (**a**) MW-PECVD setup and (**b**) the zoom-in of the growth of graphene (reprinted with permission from [96]).

It is high electromagnetic radiation of GHz range that can be used to synthesise carbon materials such as diamonds, carbon nanotubes, as well as graphene [95]. Microwave plasma (MW)-PECVD is schematically shown in Figure 12.

Chen et al. used MW-PECVD to synthesise single-crystalline hexagonal bilayer graphene (BLG) in one step with a controlled twist angle between the layers by controlling the ratio of the partial pressure of the reactive gases H_2_ (P_H2)_ and CH_4_ (P_CH4_) (P_CH4_/P_H2_) [97]. Mehedi et al. investigated an extended space of parameters as they studied the effect of growth temperature, the molar concentration of the hydrocarbon feedstock (CH_4_), deposition time and microwave power on the growth process of graphene on cobalt (Co) substrate. It was found that the process is mostly influenced by growth temperature where it significantly affected the resulted number of graphene layers leading to a minimised number of layers as well as its effect on lowering the defect density within the grown graphene film. Whilst the microwave power showed the second influential effect on the crystallinity of the produced graphene film, however, no significant effect on the number of graphene layers [98]. Thus, they showed systemically the effect of each growth parameter on the quality of the produced graphene film using MW-PECVD [98]. Furthermore, Zheng et al. reported that using ammonia (NH_3_) in the gas mixture enhanced the transforming of the vertical graphene nano-walls (GNWs) into a layer-by-layer film, compared with using the mixture of only H_2_ and acetylene (C_2_H_2_), where NH_3_ influenced long-chain hydrocarbon formation in the plasma and enhanced the etching effect. They also found that using aluminium oxide (Al_2_O_3_) substrate was better than silicon dioxide (SiO_2_) due to the low activation energy barrier of Al_2_O_3_ [96].

Electron cyclotron resonance CVD (ECR-CVD) is a modified system of MW-PECVD. It is composed of an electromagnet and a microwave system. The microwave power is connected to the plasma chamber by a bending waveguide and a circular quartz window which is surrounded by electromagnet for plasma excitation. Muñoz et al. used the ECR method to improve the direct growth of graphene films on transparent solid glasses (quartz, fused silica) at low temperature (T < 700 °C) using C_2_H_2_/H_2_ gas mixture giving bigger graphene grain size (up to 500 nm) [99]. Reports on the effect of the radicals generated in methane plasma in the ECR system [100] showed that a competition between the growth effects of C_x_H_y_ radicals and the etching effect of atomic H could take place at low temperature, suggesting that etching effect intensity of atomic H is affected by the temperature and, accordingly, affects the nucleation and growth rate. This effect of hydrogen etching by temperature was used to switch between the nucleation and the edge growth steps, by using pure methane plasma [101]. 

#### 2.3.2. Radio Frequency

Radio frequency (RF)-PECVD is a widely-used plasma source of MHz frequency, which can be coupled to plasma in three modes: the evanescent electromagnetic mode (H), the propagating wave mode (W) and the electrostatic mode (E) [95]. Al-Hagri et al. used the RF method to synthesise single-layer of vertically aligned graphene nano-sheet arrays (VAGNAs) with high surface area directly on Ge substrate at 625 °C. The synthesised graphene was tested as a surface-enhanced Raman spectroscopy (SERS) substrate and showed detection sensitivity down to 10^−6^ M of Rhodamine 6G (R6G) [102]. In addition, according to simulations and experimental results, Zhang et al. showed that silicon substrate in the RF system is surrounded by an electric sheath field that changes the local electric field in the vicinity of the substrate surface, especially on the top side of a textured silicon wafer of a pyramid nature, which leads to electric field enhancement and graphene deposition. They suggested that the different sheath electric field distribution of the pyramid structure on the bottom surface of the substrate can induce both the nucleation and growth of graphene at the same time [103]. Moreover, the effect of the growth substrate was reported by Zhao et al., comparing the growth of graphene nano-walls on glass, Si/SiO_2_ wafers and Cu foils. The morphological properties of the samples on glass substrates showed that, as the growth temperature increased, the density of vertically-oriented graphene flakes increased [104]. 

**Figure 13:**
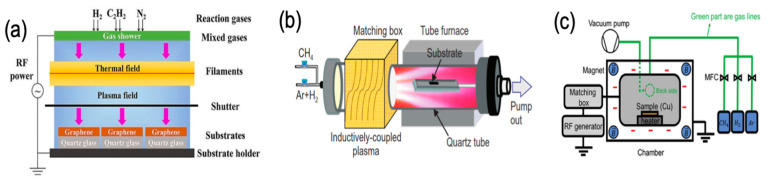
A schematic of the RF-PECVD setup accompanied with (**a**) hot filament [106], (**b**) ICP [107] and (**c**) CCP (reprinted with permission from [112]).

Combining radio frequency (RF) plasma with additional heat of a hot filament, (Figure 13a) was used to synthesise graphene directly on Ni substrate without requiring annealing step, the hot filament led to an increase in Ni grain size from nano to micro-scale and reducing the Ni grain boundaries formation which led to the growth of graphene of more ordered structure with less defects. Moreover, the high temperature eliminated oxygen contamination in the Ni catalyst and enhanced the electrical properties of the film [105]. Hot filament RF-PECVD (HF-RF-PECVD) was also used in direct growth of nitrogen-doped graphene films on glass substrate using eco-friendly N dopant by N_2_ gas instead of NH_3_ where the latter is considered as an environmental pollutant due to its corrosion ability and toxicity, and it also might cause damage to the CVD system [106]. B-doped graphene glass was also prepared by the same method using diborane (B_2_H_6_) as the B dopant.

Another modification to RF-PECVD has been made by using inductively-coupled plasma (ICP-RF-PECVD) system, where the RF power is coupled with an inductive circuit element generated by electromagnetic induction that can produce magnetic fields [93] (Figure 13b) [107]. ICP is widely used in graphene synthesis due to the high energy density and a larger plasma volume that can be produced, leading to high growth rates [95]. Pekdemir et al. investigated the effect of plasma power in the ICP system, in addition to growth time and flow rate of CH_4_ on the graphene film growth. They achieved the synthesis at 300 °C in 10 s [108]. Furthermore, Nang et al. synthesised few-layers graphene in few seconds on Cu foil and in 1 min on iron(III) oxide (Fe_2_O_3_) film. They found that increasing the ICP power and the growth time would increase the etching by atomic H, resulting in a synthesis of single-layer [107]. Rozel et al. succeeded in combining ICP-RF-PECVD with roll-to-roll technology to prepare nitrogen/silicon doped vertically oriented graphene (VOG) by using propane (C_3_H_8_) as a precursor gas and nitrogen or silane as dopants. They also found that longer deposition time caused carbon amorphisation and increased sp3-hybridized carbon fraction, resulting in expansion of vertically oriented carbonaceous and pillars growth [109].

A capacitively coupled plasma (CCP) consists of a reactor with two metal electrodes separated by a small distance [93]. Compared to ICP, CCP is a simpler apparatus setup (Figure 13c), with less energy consumption. CCP takes advantages over ICP for the lower ionisation rate of the later making most of the gases species neutrals, rather than radicals like the case of ICP. However, the voltage-drop between the glow discharge and substrate in CCP promotes ion bombardment to the grown film resulting in a formation of amorphous layer [110,111]. Yen et al. successfully prepared a single layer of graphene film of full-coverage over the substrate in less than 30 s using the CCP system [112]. The vacancy defects, which might be caused by the synthesis process of the graphene growth, was examined to adsorb gases, and it was found that the vacancy defects could promote changes of charge transfer in the graphene film with a response of about 6% under 100 ppb of NO_2_ [113].

## 3. CVD Growth Substrates

The choice of substrate is not only critical in the CVD growth process in general but turns out to be an essential parameter for graphene growth [114]. Graphene grows on different types of substrates through different processes, even within the same class of substrate material [21,115]. Historically, transition metals were the preferred substrate choice for graphene growth [21,116]. Transition metals have been studied extensively in the literature and found to produce graphene of high quality and crystallinity [21,30,115,116,117,118]. While most of the work has been done on solid metals, recent advances in liquid metals show promises in this field [90,117]. This review will investigate the recent advances in both solid and liquid metals and the critical aspects of each group of substrates.

However, before diving more in-depth in the metallic substrates, it should be mentioned that other types of materials have also been drawing scientists’ attention, especially in the semiconductor field. One drawback in metallic substrates is the potential mechanical and chemical damage caused when isolating and transferring the graphene from the substrate [117,119,120]. Thus, other types of substrates have been investigated in literature like dielectrics and silicon-based materials [30,118]. The main advantage of using such substrates is the elimination of the transfer process of graphene and the ability to grow it directly on materials used in the industrial application, making production more efficient for practical uses.

Nonmetallic substrates still fall behind metallic substrates in CVD graphene growth. A significant disadvantage is the long growth time it takes on dielectrics as well as the formation of carbides [119,121]. The use of catalysts that introduces nucleation sites on the substrate surface enhances graphene growth and provides a solution to this issue. Wu et al. grew hBN on the surface of SiO_2_ before graphene growth in situ and found that it reduced the full cover growth time to ~40 min for full coverage in comparison to tens of hours without the catalyst [119]. Another substitute is the germanium as a substrate to grow graphene. Germanium is a metalloid that does not form stable carbides and is yet compatible for desired electronic applications. Monolayers of graphene have been successfully grown on Ge (001), and the research in the field is promising. That being said, these areas of research are yet to be more thoroughly investigated before being industrially applicable [121,122,123]. 

### 3.1. Role of Substrate’s Pre-Treatment and Surface Morphology

**Figure 14:**
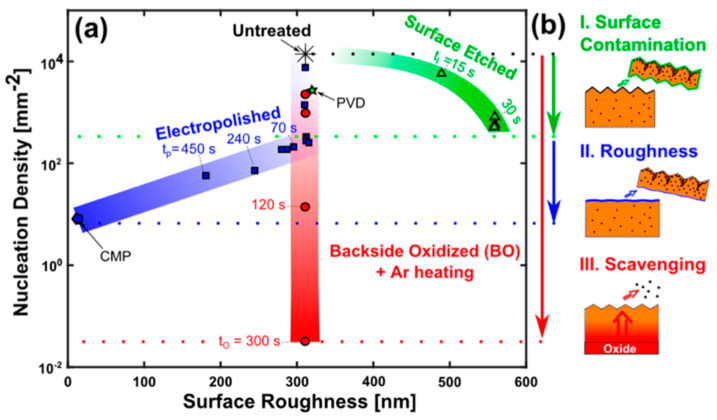
(**a**) overview of Cu pre-treatments and the effect on graphene nucleation density and Cu surface roughness; (**b**) schematic indicating the cause of the reduction in nucleation density for surface pre-treatments I−III (reprinted with permission from [125]).

Substrate’s surface morphology plays a crucial role in the nucleation and growth of graphene using CVD [124]. Thus, in order to increase the quality of the CVD grown graphene, effective control of nucleation sites is required, which can be achieved by the substrate’s pre-treatment methods. Graphene quality is profoundly affected by the size of graphene domains where larger domains are desirable; nucleation sites should be controlled in order to achieve less nucleation and larger graphene domains. Literature has discussed the role of growth substrates’ pre-treatment by introducing treatment methods such as annealing, hydrogen treatment, growth of thin films, plasma etching, chemical etching and electropolishing (Figure 14). Those methods showed an effect in modifying the substrates’ morphology such as its surface roughness, grain size, impurities density, native oxide thickness, etc., which all lead to control the active nucleation sites, thus, more homogenous and uniform graphene deposition.

The quality of the deposited graphene was reported to be highly improved and become more uniform by using a Cu substrate with a thin layer of evaporated Cu on the surface (200 nm). This suggested that the precise evaporation of this Cu thin film enhances the smoothness of the growth substrate that reflects on the uniformity of the produced graphene [126]. Moreover, it was shown that the substrate’s orientation and pre-annealing strongly affect the thickness uniformity of the grown graphene [127]. It was claimed that forming a smooth Cu (111)-oriented surface during pre-annealing leads to the formation of monolayer graphene with improved uniformity and nearly perfect centimetre-scale samples [127]. Another substrate treatment that modifies surface morphology is using nitric acid for etching the as-received Cu foil using short exposure time (30 s) showing that it improves the quality of the produced graphene film [128]. The effect of using nitric acid was also reported by Kim et al. when Cu foil was annealed first under the flow of hydrogen, followed by etching using nitric acid. It was found that pre-annealing under H_2_ removed the native Cu oxide where the nitric acid removed the rolling lines and impurities, providing a smoother surface for graphene deposition. It was found that pre-treated Cu foil exhibited 98.3% of the grown graphene as monolayer compared to 75% of monolayer graphene on untreated substrates [129].

Nitric acid was also used by several groups for an effective substrate’s pre-treatment. Lee et al. have used different exposure times of Cu substrate immersed in nitric acid and studied its effect on the substrate morphology and the grown graphene [128].

**Figure 15:**
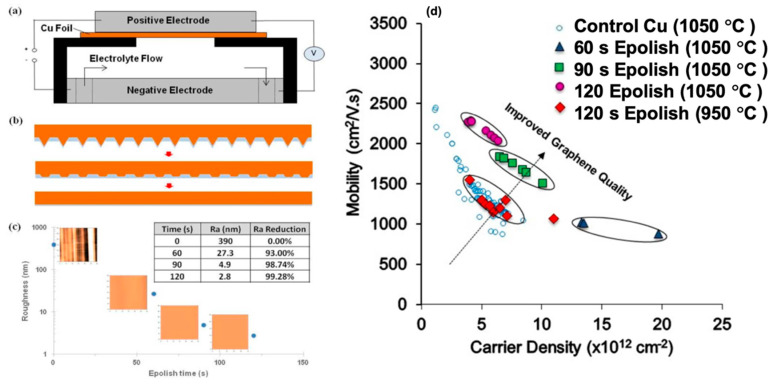
Schematic of (**a**) EP setup and (**b**) planarization mechanism during polishing. (**c**) AFM images showing as received Cu compared to EP Cu using different EP times. The inset table displays the measured surface roughness of the EP samples over time and the reduction rate from the as-received sample (reprinted with permission from [130]); (**d**) a plot of mobility versus carrier density of CVD grown graphene on EP Cu (reprinted with permission from [131]).

Electropolishing (EP) has also been reported as an effective pre-treatment to achieve smoother substrate (Figure 15a–c). However, many involved variables directly affect the EP process, such as the used electrolyte (composition, viscosity), EP setup (stirring speed, temperature, sample shape, electrodes, voltage, etc.). Griep et al. developed a modified EP method [130,131] that led to a drastic reduction of Cu surface roughness of 99%. This enhancement in smoothness decreased the formation of BLG defects, a reduced sheet resistance of MLG (120 Ω/cm^2^), altered domain sizes, and 78% higher breaking strength [130]. It was also shown that enhanced surface smoothness directly affected the mobility of CVD grew graphene by 125% compared to the untreated substrate in addition to lower planarisation level providing insight on the effect of substrates’ smoothness on the grown graphenes’ electrical properties (Figure 15d) [131]. 

**Figure 16:**
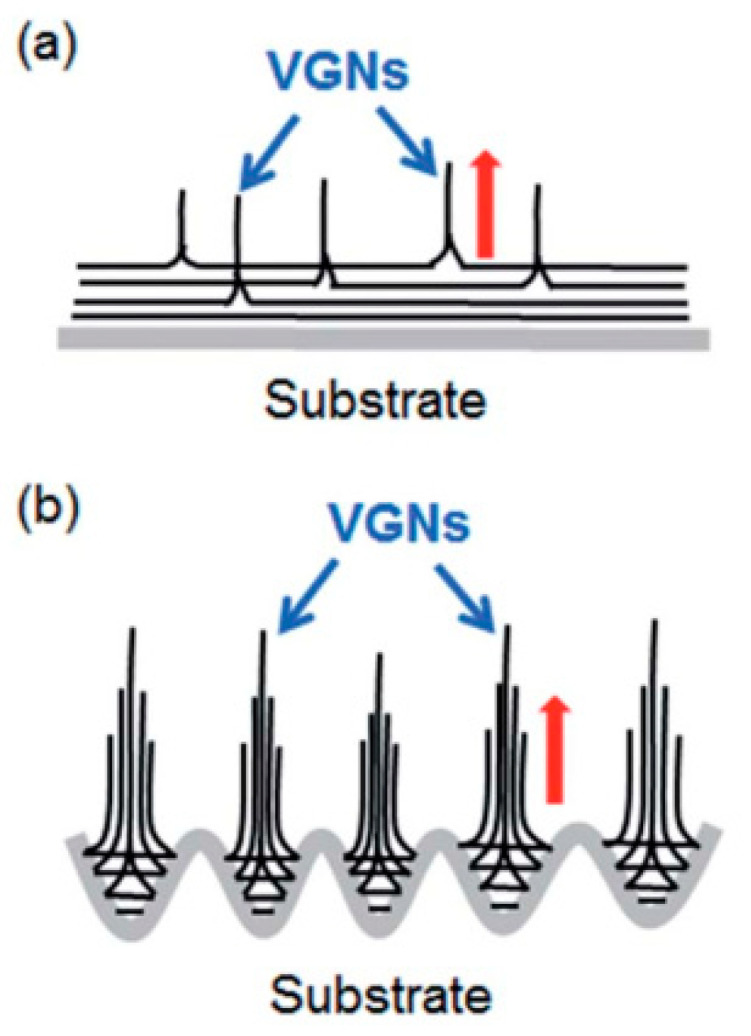
Scheme for the mechanism of VGN growth on (**a**) untreated and (**b**) Ar plasma-treated substrates (reprinted with permission from [132]).

In addition, the effect of substrate’s treatment using Ar plasma was studied by Sui et al. reporting its effect on the growth of vertically oriented graphene (VOG) via PECVD involving etching by hydrofluoric acid (HF) and oxidation by oxygen gas after Ar plasma treatment. They showed that carbon precursors are slightly adsorbed on the untreated substrate forming planar nanoflakes with less probability for the growth of VOG. However, Ar plasma-treated substrates can create a microcavity on the substrate surface, which are considered to be active growth sites. According to Stranski–Krastanov growth model [132]. The strain accumulation of VOG is decreased on the rough surface of Ar plasma-treated substrates, resulting in the transformation of 2D planar films to the 3D cluster, as illustrated in Figure 16. Oxidation of the substrate after HF etching resulted in more dense formation of VOG due to the passivation of Ar plasma by the oxidation.

**Figure 17:**
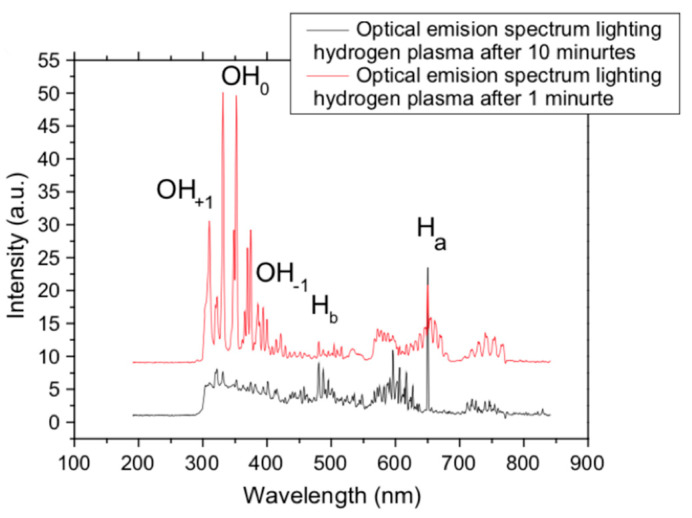
OES of the Cu reduction after 1 and 10 min of the exposure to hydrogen plasma, showing the reduction of the OH peaks that indicates the reduction and removal of the native oxide layer on the surface of Cu substrate (reprinted with permission from [77]).

In another study of plasma etching as a pre-treatment method for the growth substrate, Chaitoglou and Bertran used hydrogen RF plasma etching of 100 W at a pressure of 10 Pa for 10 min to remove the oxide layer on the Cu substrate [77]. The characterisation was conducted after the substrates’ pre-treatment by using the optical emission spectroscopy (OES) analysis method. The reduction of the OH radicals peak after being exposed to hydrogen plasma etching indicates the reduction of the oxide layer until the peak was disappeared, showing that no more oxide atoms were available (Figure 17) Thus, the hydrogen plasma etching after such a short time of exposure demonstrated its effectiveness on removing the native oxide layer from the Cu substrate.

Furthermore, Murdock et al. investigated the effect of the contamination present on the Cu foil surface of different purities [133]. They reported the presence of Ca, Al and Si contamination in the commercial samples, and by investigating varying purities of Cu substrates, it was found that the supplier-stated purity did not show a specific trend in the quality of the graphene growth. It was rather related to the impurities present (in the range of few nanometers) and their distribution. Murdock et al. used chemical treatments (HCl, KOH, HF, HCl/KOH) to clean the substrates which were effective in removing the impurities in the Cu foils. 

### 3.2. Solid Substrates

Transition Metals have shown leverage over other materials in CVD graphene growth. Their catalytic feature makes them more favourable than other materials. Transition metals including Ni, Cu, Pd, Ru, Ir and Pt have been studied extensively for single layer graphene growth using CVD; the two most commonly used substrates are Ni and Cu [115,116,134]. In fact, formation of graphene layers on Ni dates back to more than 50 years where it was observed in industrial applications [21,134,135]. Ni(111) has the least lattice mismatch with graphene compared to other transition metals (<1%) making it catalytically more favourable for growing graphene [136]. Graphene grows on Ni through bulk diffusion [21,115,118,136]. In this process, carbon atoms diffuse into the bulk of the substrate due to the solubility of Ni. Upon cooling, segregation occurs and C atoms precipitate on substrate’s surface forming the graphene layers. Grain boundaries and defects on the substrates surface increase the nucleation sites, which lead to more heterogenous graphene growth [115,118,135,136].

**Figure 18:**
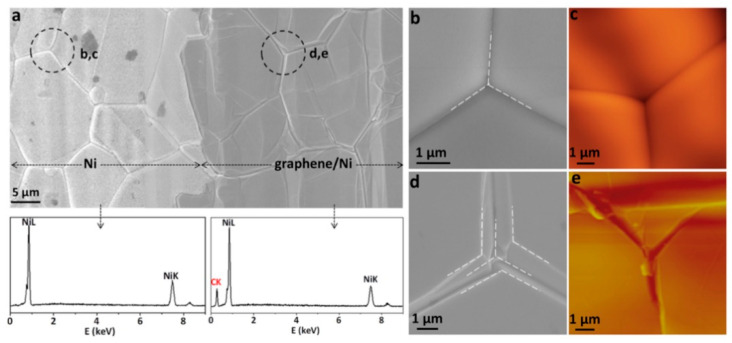
(**a**) FESEM image recorded at the interface of Ni (left) and graphene/Ni (right). The absence and presence of graphene in two regions are confirmed with the EDAX spectra shown below them. The circles represent the scenarios in (**b**) and (**d**) where grain boundaries meet. A FESEM and AFM image of annealed Ni foil (**b**,**c**) and graphene peeled-off Ni foil (**d**,**e**). z-scale: 1 μm. Dashed lines are drawn for easy guide. (reprinted with permission from [136]).

Ani et al. provided an extensive review of the chemical and physical factors affecting the nucleation sites and argued for the advantage of using single crystalline (both Ni and Cu) to produce higher quality graphene [115]. However, researchers tend to favour developing the use of polycrystalline Ni for its economic advantage [135,136,137]. Mogera et al. investigated twisted multilayer graphene growth on polycrystalline Ni [136]. It was found that graphene growth on polycrystalline Ni enhances Ni(111) orientation when compared with other treatments of Ni including annealing. Figure 18 shows the difference between the interlock of the sample with and without a graphene layer. The more complex junction shown in the graphene/Ni grain boundaries results from the multi-stacking of graphene layers which induces (111) morphology in the polycrystalline Ni. Zou et al. also reported on the effect of CVD graphene grown on the underlying layers of polycrystalline Ni [137]. By observing the graphene growth on Ni(100) crystal steps, Zou et al. reported the possibility of growing single graphene crystals even in the presence of bunch steps in the substrate where the graphene grown smooths the substrates surface by introducing monoatomic steps to overcome the bunch step. Research in polycrystalline substrates at the atomic scale sheds the light on the physical process at the C-Ni interface which will be useful in bringing polycrystalline Ni to practical applications. 

**Figure 19:**
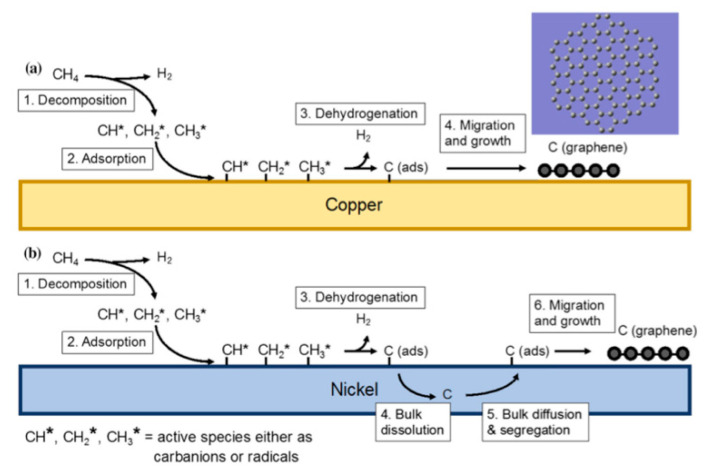
Difference in graphene growth processes between (**a**) Cu and (**b**) Ni substrates. Surface diffusion occurs on Cu substrate while bulk diffusion and precipitation occurs on Ni (reprinted with permission from [115]).

Experimenting with different metal substrates, copper soon raised as an excellent substrate for CVD growth of Graphene [38,91,138,139,140]. Early work by Li et al. found that the low C solubility of copper at high temperatures reduces precipitation on the surface and results in more uniform single-layer graphene growth [38]. Figure 19 demonstrates the difference in the processes of graphene growth between Ni and Cu. It is found that graphene growth on Cu occurs by surface adsorption of C atoms over the copper surface. Copper has stable electronic configuration which limits its reaction with C atoms to weak forces and reduces the bulk diffusion of C atoms in Cu substrate [21]. This gives copper an advantage in growing single layer graphene, where the process progresses on the surface [21,139]. 

While the surface diffusion of reaction limits the graphene growth to single layer upon coverage of the copper surface, it has been shown that more layers can be grown on copper [51,52].

In brief, the graphene growth mechanism on catalytic metal substrates can be summarized when the CH_4_ decomposition in the CVD reactor is very complicated with the possibility of many possible chain reactions. CH_4_ can react via pyrolysis or chain reactions in the gas phase even without a catalyst if the temperature is sufficiently high to aid thermal decomposition and when P_CH4_ is high. Gas phase reactions provide CH_3_, H_2_, H and C_2_H_x_ (x = 1–6) [14]. In the case of Cu, CH_4_ is believed to thermally and catalytically decompose on the substrate since Cu is a known dehydrogenation catalyst. This catalytic decomposition process starts with the absorption of the CH_4_ molecule and ends with a final product of one carbon atom and four hydrogen atoms on the surface of the Cu substrate via three intermediates: methyl (CH_3_), methylene (CH_2_) and methylidyne (CH) [141]:

CH_4_ → CH_3_* + H

CH_3_ → CH_2_* + H

CH_2_ → CH* + H

CH → C + H

In these dehydrogenation reactions over Cu, the CH monomer dissociation is difficult to complete and is considered as a rate-limiting step. The formation of dimers occurs when the CH monomers on Cu continue their path with simultaneous dehydrogenation reactions [142]. According to first principles calculations within density functional theory (DFT), CC dimers were found to be stable on all sites on the Cu surface [30]. Moreover, carbon dimers containing hydrogen are very undesirable on low-energy surfaces, even on defects, as they desorb or immediately decompose even at low temperatures. This has been proven by using temperature-programmed desorption (TPD) and thermal desorption spectroscopy (TDS) [143]. 

Two further analyses can be conducted to obtain a more detailed understanding of the process: kinetic analysis and thermal analysis. Kinetic analysis considers the progression of the various processes over time, which may occur in the gas phase or on the growth substrate. Thermodynamic analysis is time-independent and considers the lowest equilibrium energy configuration of a system under isothermic and isobaric conditions, hence, defining the limits of the parameters which may successfully be used for deposition. 

Firstly, the hydrocarbon species are diffused through the boundary layer to reach the substrate’s surface. In the case of adsorption, the hydrocarbon species are adsorbed on the surface, followed by decomposition in order to provide the required carbon species to be diffused on the surface or in the bulk of the used substrate, depending on the substrate’s carbon solubility. The inactive hydrogen species are finally diffused as a by-product through the boundary layer back into the main gas stream. These steps are mainly classified into two scenarios/categories, the mass transport region where the diffusion through the boundary layer takes place, and the surface reaction region [45]. In addition, it has been reported that, when using low-energy electron microscopy (LEEM), there was no precipitated carbon or isolated growth of graphene islands during the cooling step in the case of using Cu substrate [144]. This agrees with the conclusion whereby graphene deposition on Cu is limited to surfaces showing no carbon precipitation from the substrate.

Interestingly, it is found that the thickness of the substrate has an effect on the number of graphene layers grown on the substrate. By growing graphene on several thicknesses of Cu substrates, Yilmaz and Eker found that increasing the substrate’s thickness decreased the grain size of the substrate [145]. Looking at a thickness range of 9–250 µm, the results showed single-layer graphene was grown on all substrates, but the 150 µm particularly stood out giving the best quality graphene; the other substrates had few-layer graphene as well. While the authors showed the difference with increasing thickness, it was not clear why 150 µm substrate had the best results (instead of 250 µm). The authors did point out that increasing thickness in the substrate also corresponded to decreasing micro-strain which they reported to be essential for single layer graphene growth. Nguyen et al. used a quartz cap over the substrate to reduce impurities which, as they reported, also emerged from the bulk of the substrate [146]. Huet et al. also suggested that the formation of round-shaped and branch like multilayer graphene is a result of substrate’s bulk reaction [52]. 

### 3.3. Liquid Substrates

Whilst the use of solid substrates is common, liquid metals emerge in the field as an excellent substrate choice for CVD grown graphene. The surface tension and thermal motion of liquids provide a “random closed-pack” geometry [90,118]. Thus, liquid substrates do not exhibit grain boundaries which have a significant effect on the graphene quality grown on solid substrates [90,118,147,148]. Liquid substrates also allow higher surface diffusion rate of carbon atoms due to the weak interaction with the surface and lower migration energy [118,148]. The surface of the liquid substrate exhibits rheological features allowing self-assembly of graphene crystals on the substrate [90]. In Deng et al. review on a mass production of graphene film, it is found that graphene growth on liquid Cu offers excellent control over the layer quality [147]. 

Zeng et al. investigated several liquid metal substrates (In, Ga and Cu) under varying growth conditions [149]. This study gave one of the earliest insights on graphene growth over liquid metal substrates. Comparing the different liquid metals shows that it is the liquid properties that give superiority in growing homogenous single-layer graphene rather than the different metal qualities.

**Figure 20:**
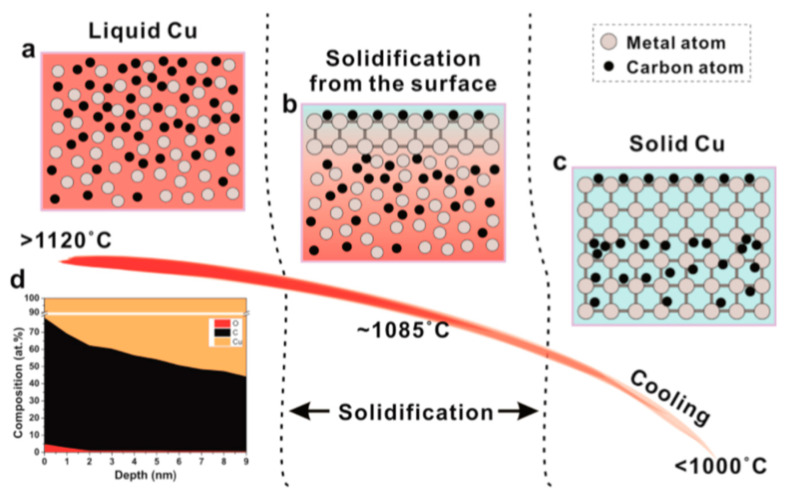
Schematic illustration of the carbon distribution in (**a**) liquid Cu, (**b**) solidified surface of the liquid Cu and (**c**) solid Cu. (**d**) XPS composition profile showing elements (O, C and Cu) along the surface after CVD graphene growth showing a larger amount of C dissolved in bulk, demonstrating the increase in carbon solubility in Cu at its liquid state (reprinted with permission from [149]).

Liquid substrates possess a uniform amorphous structure that allows the growth of sizeable single-layer graphene. Figure 20 demonstrates the graphene film growth process on liquid Cu substrates and shows an interesting difference compared to growth procedure on solid Cu substrate shown earlier in Figure 19. As it can be seen, the C atoms diffuse into the liquid bulk of Cu in a similar behaviour to the one observed on solid Ni. However, upon cooling, the precipitation of the C atoms in the bulk is prevented by the solidified surface of the substrate, resulting in a single layered graphene film. It has been observed that the formation of the graphene film withstands large variations in growth conditions compared to solid substrates, which gives it another advantage. It has also been observed that graphene grown on solid Cu substrate can be transformed to more homogenous film by melting the Cu substrate [149,150]. Such feature can be used to grow graphene layers that are both continuous and homogenous taking advantage of both solid and liquid substrate qualities [150].

Another study by Wang et al. found a similar ability in withstanding varying conditions in earlier work where graphene films were grown on Ga [50]. Even when an excess of methane was provided, graphene film maintains a single layer formation without multilayers, indicating a self-limiting process. Also using Ga substrate, Lu et al. developed a novel sliding transfer method for graphene films without loss of integrity or damaging the graphene films [151]. The delocalised atomic structure of liquid metal substrates gives it other leverage over solid substrates when it comes to transferring the graphene film from the substrate. 

However, it is found that liquid substrates can be used to grow multilayer graphene which also has important applications in electronics and spintronics. Ma et al. successfully grew AB-stacked bilayer graphene using a substrate of solid Pt with a top layer of liquid Pt_3_Si [152]. The growth mechanism was studied using ^12^CH_4_ and ^13^CH_4_ carbon isotopes. It is seen that C atoms diffusing into the solid Pt precipitate through the liquid layer upon cooling and form a second layer of graphene beneath the previously formed layer. The lattice of the second graphene layer was also found to be affected by the lattice of the first layer. This phenomenon of interlayer epitaxy opens the floor of controlled multilayer graphene growth using liquid substrates. 

As it was aforementioned that process conditions such as tailoring the hydrogen and hydrocarbon flow rates directly affect the resulted graphene film, in the reported study (previously mentioned in Section 2.1.2), Wu et al. showed that graphene flakes morphology can be controlled by tailoring the gas flow rates (Ar, H_2_ and CH_4_) [63]. The correlation between tailoring the gas mixture and the properties of the liquid substrate can be discussed as follows. The presence of CH_4_ molecules on the surface of the substrate lowers the C atoms mobility by creating energy barriers. When the Ar:H_2_ ratio is lowered (up to where only hydrogen was used), adsorbed C atoms mobility is lowered on the substrate surface, C atoms diffuse along the edges of the island to find a favourable energy location and form the round shaped flakes. While increasing Ar:H_2_ ratio and hence reducing CH_4_ molecules on substrates surface favours surface diffusion along substrate over bulk diffusion along islands edges, resulting in a dendritic shape. This flexibility of controlling flake shape by varying gas mixture is supported by the properties of liquid Cu that ease adsorbed particles diffusion on the surface. CVD growth of graphene on liquid metal substrates is an area surely to be investigated further in the future.

The review of the graphene growth on CVD suggests that substrate choice is a key factor affecting the quality of the graphene grown. Different substrates react differently, resulting in a varying growth time and product quality. While pre-processing, annealing and longer growth times have been found effective in improving graphene quality, a middle ground is to be found between the quality and processing time to make CVD grown graphene economically viable.

A summary of CVD graphene growth recipes including the process parameters is in Table 1.

## 4. Characterisation of CVD Graphene

### 4.1. Observation of Graphene Grain Boundaries

The quality of CVD graphene is highly affected by the unavoidable defects during growth processes. Defects such as impurities, vacancies and grain boundaries can be present in CVD graphene sheet; however, grain boundaries have the biggest influence on the resulted graphene quality [172]. Therefore, the visualisation of graphene’s grain boundaries is very beneficial when post growth is conducted and prior to more detailed characterisation. Typically, techniques such as transmission electron microscopy (TEM), scanning tunnelling microscopy (STM), optical microscopy, liquid crystal deposition and oxidation are used for the observation of grain boundaries [173,174,175,176,177]. Kim et al. utilised TEM to provide an efficient way to visualise polycrystalline grain boundaries [174]. Scanning electron diffraction in STEM and dark field (DF) TEM imaging techniques were proved to be fast and direct methods for observing CVD graphene’s grain boundaries. In SED-STEM, nonparallel electron beam was used where gradual transition were observed reflecting rotations in sets of grain boundaries. In addition, DF-TEM could identify facile highly rotated grain boundaries (>~10°) as a change in image contrast that clearly shows lines representing grain boundary. Combined SED-STEM and DF-TEM enabled the mapping of the obtained grain boundaries of CVD graphene. They marked the locations where the diffraction patterns made transitions when using SED-STEM to create a map and DF-TEM confirmed the grain mapping [174]. Another approach was presented by Fan et al. to visualise grain boundaries after graphene transfer on SiO_2_ substrate by exposing graphene to vapour hydrofluoric acid (VHF) [177]. The VHF has an etching effect on SiO_2_ and upon vapour exposure on graphene, VHF diffuses through the graphene grain boundaries to release the substrate and partially etch it. Therefore, grain boundaries can easily be seen by tracing the etching marks using a simple optical microscopy. An alternative method for detecting graphene domains was reported by Son et al. using the liquid crystal layer [178]. The graphene sheet was grown using CVD and then transferred on the glass substrate. Then, droplet of liquid crystal mixture was placed on graphene. The alignment of liquid crystal molecules over the graphene sheet was found to be strongly correlated with graphene’s domain size. It was found that the anisotropically alignment of liquid crystal molecules with respect to graphene domain exhibit distinct birefringence nature that allow a direct way to image and identify graphene domains/grain boundaries. Moreover, simple and direct visualization of grain boundaries was demonstrated by Kunka et al., showing that it can be conducted using straightforward room temperature oxidation of CVD grown graphene on Cu substrate [179]. Graphene provides an excellent layer to protect the underlying growth metal substrate from oxidation even at elevated temperature. However, defects and grain boundaries in graphene layer allows the diffusion of oxygen molecules to reach the underlying metal and cause oxidation. Their study showed that oxidation of polycrystalline copper substrate can be confined by graphene’s grain boundaries. 

The visualisation of grain boundaries in CVD graphene is important to efficiently evaluate the grain boundary density on large-scale graphene samples. This provides a simple and useful method to speed up the process of developing the synthesis of large-scale and high quality graphene.

### 4.2. Raman Spectroscopy

Raman spectroscopy is defined as a non-destructive, fast and non-invasive spectroscopic technique where a laser source interacts with a sample, causing inelastic scattering of monochromatic light and a structural fingerprint by molecules can be identified. The aim of using Raman spectroscopy is to analyse the Raman scattered light and infer from it as much as possible information about the chemistry and structure of the material. 

**Figure 21:**
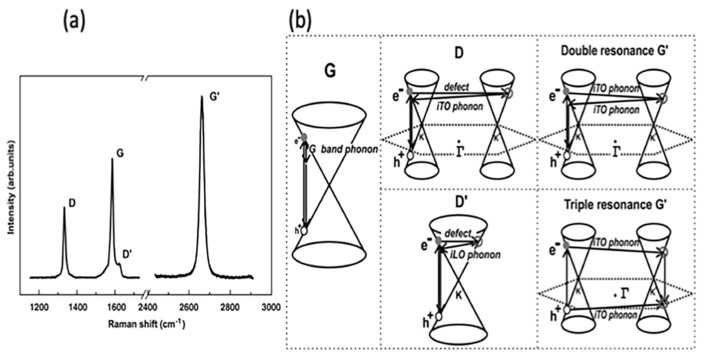
(**a**) characteristic Raman spectra of graphene and (**b**) Raman scattering processes (reprinted with permission from [186]).

Raman spectroscopy has a long history of being considered a powerful technique for the structural characterisation of graphitic materials [180,181,182,183]. G-bands and 2D-bands (the latter also known as the G’-band) are the two distinct Raman features in almost all crystalline sp^2^ materials. The G-band is a first order scattering which occurs due to the bond stretching of all the pairs of sp^2^ atoms and is positioned at 1583 cm^−1^ whilst the 2D band (G’-band) is a second order scattering positioned at 2670 cm^1^, and the D band is a third characteristic peak at 1350 cm^−1^ [184,185]. The double resonant processes in the 2D and D bands occur between the non-equivalent K points in graphene’s first BZ. In the case of the 2D mode, the scattering connects two zone-boundary phonons while the D mode connects a single phonon and a defect. Thus, the D band is absent in high-quality graphene that is defect free. The G* mode occurs from a combination of the zone-boundary in-plane longitudinal acoustic phonon and in-plane transverse optical phonon modes [185], characteristic Raman peaks are shown in Figure 21a.

The order of a Raman band is defined by the number of scattering events involved, e.g., the G band is first order, whereas the 2D band (two phonons) and D band (one phonon and a defect) are second order. A final class of categorisation for graphene materials is that the scattering process can be categorised as an intravalley (AV). AV scattering occurs around the same high-symmetry K-point in the BZ [186], while the other scattering category known as intervalley (EV) occurs where two inequivalent high symmetry K and K^’^ points are involved (Figure 21b).

In graphene with Bernal stacking, the Raman spectrum is dependent on the numbers of layers; thus, Raman provides a facile method through which to measure flake thickness, albeit with caution around the other factors which may affect the spectrum [185]. 

**Figure 22:**
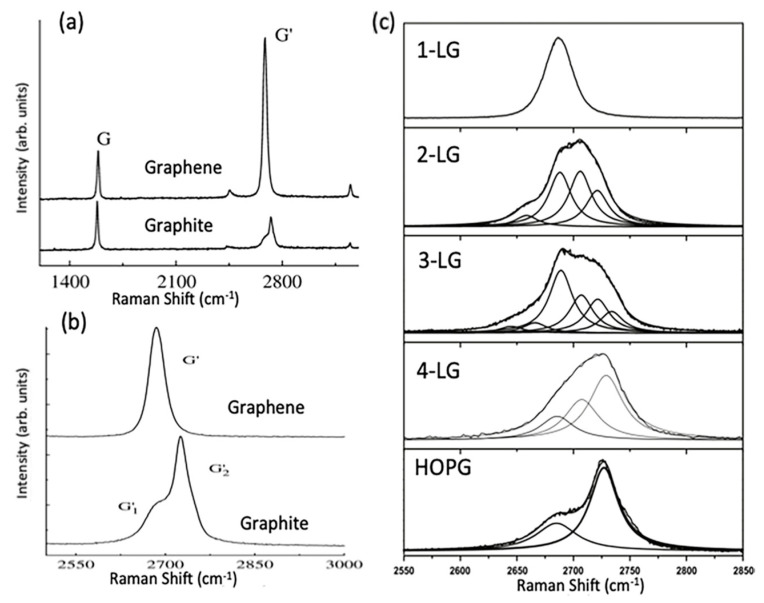
(**a**) comparison of the Raman spectra of graphene and graphite using a 514 nm excitation wavelength. (**b**) a close-up comparison of the 2D peaks in graphene and graphite (reprinted with permission from [184]); (**c**) measured 2D Raman band of different graphene layers showing the splitting of the 2D band which opens up as it goes from 1-LG to 3-LG and then closes up as it goes from 4-LG to highly oriented pyrolytic graphite (HOPG), (514 nm excitation wavelength) (reprinted with permission from [186]).

**Figure 23:**
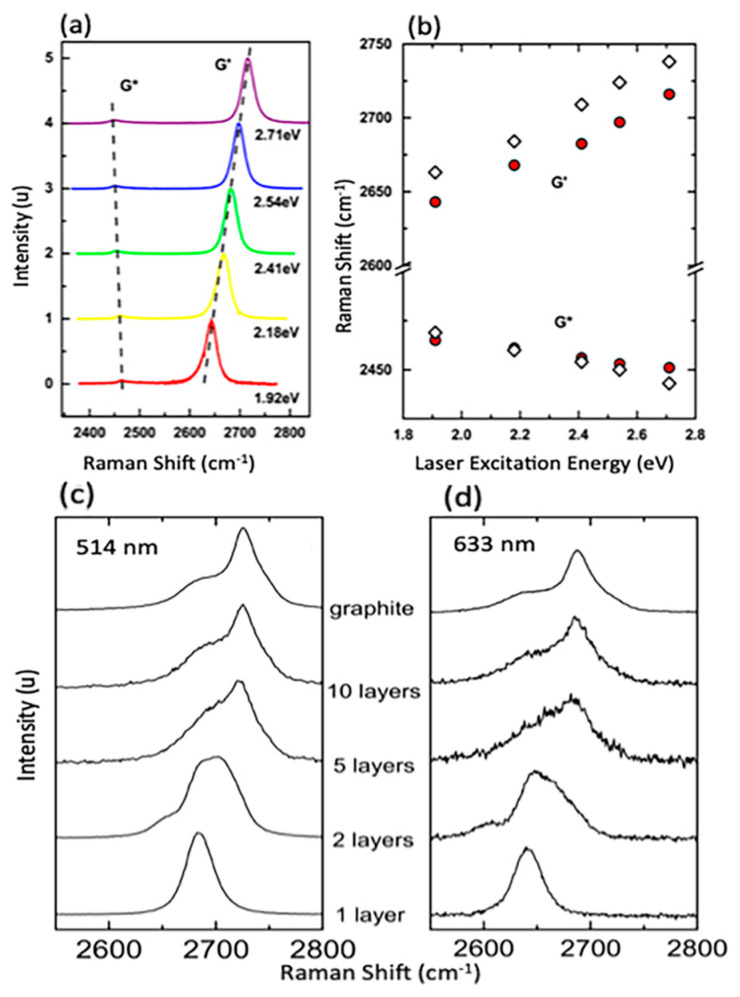
(**a**,**b**) comparison of the Raman spectra of G^’^ (2D) and G* bands of monolayer graphene showing linearly blue shifts of the G’ band with increasing excitation energy (the circles correspond to graphene, and the diamonds correspond to turbostratic graphite); (**c**,**d**) the change of the 2D peak shape as a function of the number of graphene layers are shown for 514 and 633 nm excitations, respectively (reprinted with permission from [184,186]).

When the number of layers increases, 2D peak intensity reduces and becomes broader and upshifted in comparison to monolayer graphene. In addition, the difference in the 2D shape between graphene and graphite is very noticeable. This difference is due to the fact that, in graphite, the 2D mode consists of two components, 2D_1_ and 2D_2_, which are about ¼ and ½ the height of the G peak, respectively. However, graphene has a unique single, a sharp 2D peak which is roughly four times the intensity of the G peak. The splitting of the band structure in Bernal multilayer graphene means that there are more electronic levels accessible for each double resonance event, each contributing extra components to the 2D band. Two of the four electronic transitions near the Dirac points can be coupled with optical excitation as demonstrated by density functional theory [187]. For each transition, the electron can scatter from one of two phonons [188] leading to a four component 2D band. (The components are split over ~70 cm^−1^, and each has FWHM of 24 cm^−1^ [187].) The G* band for three-layer Bernal graphene has been empirically shown to be comprised of six Lorentzian peaks with an FWHM of 24 cm^−1^ splits over ~130 cm^−1^ [186]. Theoretical analysis suggests that as many as 15 different phonon frequencies can contribute to the G* band, although the separation between modes is often below the resolution of the spectrometer [186]. Adding more graphene layers smears the accessible phonon frequencies further, such that they can no longer be distinguished from each other. Once a flake is thicker than five Bernal layers, a broad, dual peak of 3D graphite is seen near the G* frequency [187]. Bernal stacking of successive graphene layers opens a wider range of frequencies for G* scattering events. Hence, the maximum intensity of the band is lower than that for monolayer graphene (Figure 22a). Integrated intensities of G and D peaks remain approximately the same [189]. Another important factor that influences the position of the 2D Raman band is the choice of laser excitation energy (Figure 23a,b). The excitation energy also affects the shape of the 2D band as a function of the number of graphene layers, as demonstrated in Figure 23c,d. G band to 2D band intensity increases with the number of graphene layers present [184,187]. 

#### Graphene Disorder in Raman Scattering

**Figure 24:**
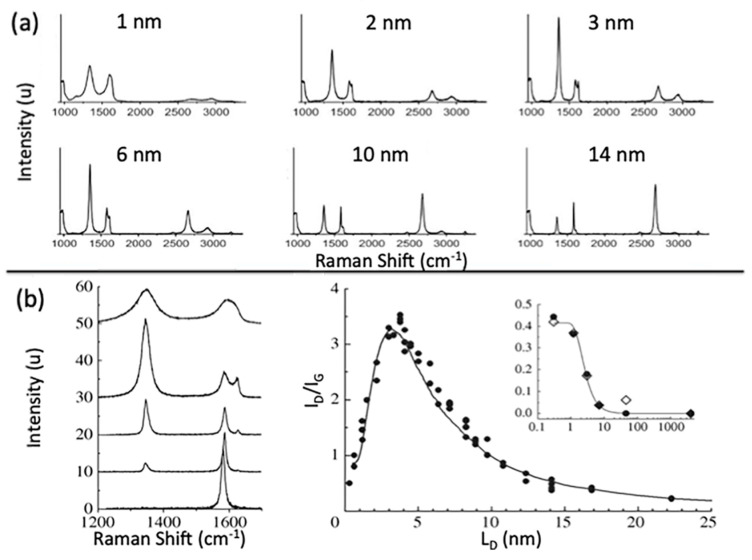
(**a**) illustration of Raman spectra of graphene oxide (GO) as a function of the distance between defects (reprinted with permission from [192]); (**b**) the ratio of I_D_/I_G_ from different monolayer graphene samples as a function of the average distance L_D_ between defects that are induced by the Ar^+^ ion bombardment, the inset ratio of I_D_/I_G_ versus L_D_ for two graphite samples (reprinted with permission from [193]).

As aforementioned, D-bands need defects, impurities, or grain boundaries to be activated and appear in spectra. Thus, a D peak is absent in high-quality graphene as no defects are present to break the symmetry. The Raman scattering cross-section of the D band is known to be proportional to the concentration of defects. In the early 1970s, it was first demonstrated that the intensity ratio I_D_/I_G_ relates to average grain size, but this relation was limited to bulk graphite and one laser excitation energy (514 nm) [180]. Almost 30 years later, in 2006, the work was extended to different laser excitation energies, and I_D_/I_G_ was found to be inversely proportional to the fourth power of the laser excitation energy (E_L_) [190], as described in Equation (1):

(1) $${\left(\frac{I_{D}}{I_{G}}\right)}^{-1}=L_{a}\frac{E_{L}^{4}}{560}$$

where L_a_ is the grain size measured in nm, E_L_ is the excitation laser energy and is given in eV, and the constant 560 is given in units eV^4^/nm. Later in 2010, experiments were conducted to study a graphene disorder caused by Ar^+^ ion bombardment at low energy (90 eV) [191]. Through observing the I_D_/I_G_ ratio change with different doses of ion bombardment, it was possible to quantify the density of defects using highly oriented pyrolytic graphite (HOPG) for calibration. The relation between defects density and I_D_/I_G_ ratio is described using Equation (2):

(2) $$\frac{I_{D}}{I_{G}}=C_{a}\frac{r_{a}^{2}-r_{s}^{2}}{r_{a}^{2}-2r_{s}^{2}}\left[\exp\left(-\frac{{\pi r}_{s}^{2}}{L_{D}^{2}}\right)-\exp\left(\frac{\pi \left(r_{a}^{2}\right)}{L_{D}^{2}}\right)\right]$$

where C_a_, r_a_ and r_s_ are adjustable parameters determined by experiments, and L_D_ is the distance between defects. The model measures the change in the two length scales for graphene sheets caused by a single ion; the radius r_a_ is of the circle of the activated incident ion impact point where r_s_ is the radius of the circle of the structurally-defective ion beam. The relation between the ratio I_D_/I_G_ is demonstrated in Figure 24.

In summary, Raman spectroscopy reveals a tremendous amount of information about the properties of graphene by probing the interplay between its electronic and vibrational structures. Several graphene layers, film’s uniformity, doping effect, defects nature and defects density, domain size, etc. are all possible through the analysis of graphene’s Raman mapping and spectra, hence, a tremendous essential tool in graphene research.

### 4.3. Scanning Electron Microscopy

Scanning electron microscopy (SEM) is one of the most powerful and frequently used imaging techniques in materials research and graphene characterisation in particular. In graphene research, SEM is frequently used to characterise graphene grown on conductive substrates using the CVD method. It provides valuable information about graphene growth rates, sample coverage, nucleation density, grain size and morphology, but it cannot determine the exact number of graphene layers, providing only an estimate of the graphene layers’ uniformity. However, the contrast in SEM images reveals qualitative information about the thickness of the deposited graphene, where darker parts are covered with a larger number of graphene layers and lighter parts are covered with less. Thus, areas of more uniform contrast indicate a better graphene film coverage with more homogeneous graphene thickness and layer numbers. This contrast is due to the amount of secondary electrons that are generated in the upper few nanometers of the sample surface [56]. In addition, SEM has advantages in imaging impurities and defects within graphene sheets, such as folds, voids and film breakage of as-grown or transferred graphene. Investigating graphene quality is essential for applications, and low voltage field emission SEM (FESEM) is a possible way of achieving this.

**Figure 25:**
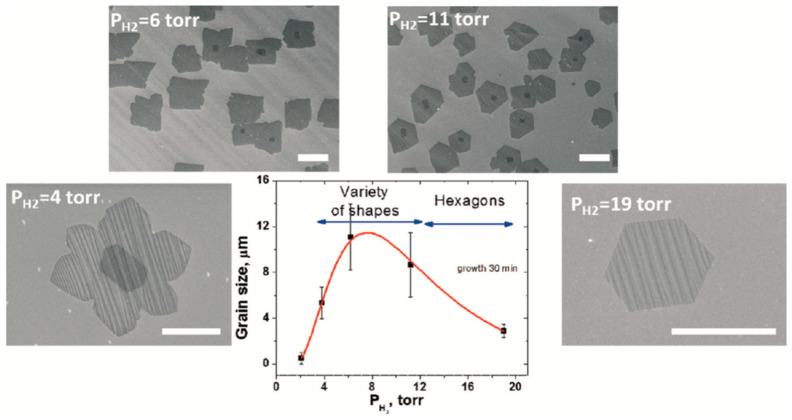
SEM images showing the morphology of the obtained graphene domains as a function of hydrogen partial pressure and its effect on grain size. Scale bars are 10 µm (top two images) and 2 µm (bottom two images) (reprinted with permission from [61]).

**Figure 26:**
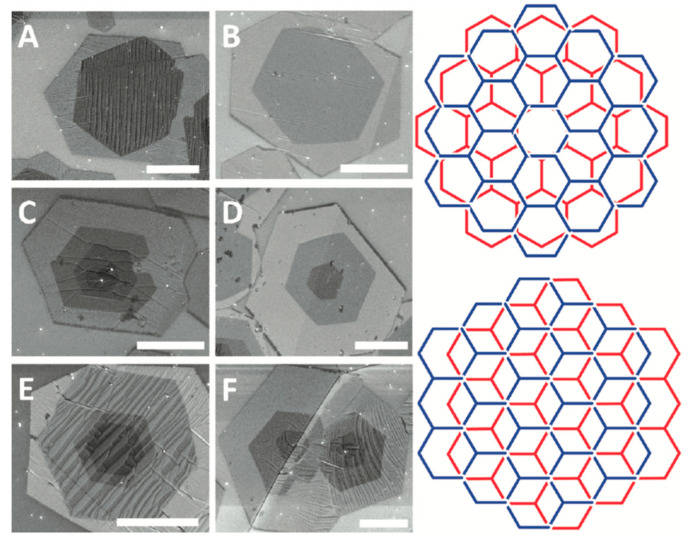
Analysis of mutual orientation between the layers in multi-layered graphene grown for 30 min at 60 ppm CH_4_, 19 Torr hydrogen pressure. All layers have hexagonal shapes in distinct contrast to irregular grains at higher methane concentrations. The second layer often appears misoriented with respect to the first layer, frequently showing 30 degrees rotation (right graphic) (**A**,**B**,**E**), while some do show what resembles AB Bernal stacking (**C**,**D**). The third and fourth layers, on the other hand, always show AB stacking (**C**,**F**). Scale bars are 3 µm (reprinted with permission from [61]).

Vlassiouk et al. reported the crucial role of hydrogen concentration on the produced CVD graphene. The results were presented using different characterisation techniques; however, SEM was the primary tool to demonstrate the graphene shapes, grain sizes and nature of the edges and orientation as a function of hydrogen partial pressure (P_H2_) [61]. The SEM images in Figure 25 clearly shows that the morphology of the graphene domains varied with P_H2_, giving irregular mixed shapes at lower P_H2_ with some of the 6-fold domains, while nearly perfect hexagons are achieved at higher P_H2_. SEM images could also clearly show rotations of the graphene domains and their orientations that also provides information about the stacking of the graphene layers (e.g., AB stacking, as shown in Figure 26). 

**Figure 27:**
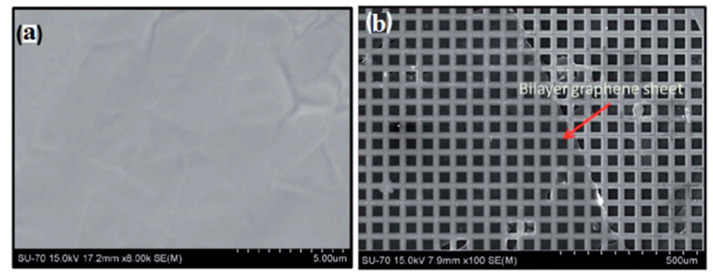
(**a**) FESEM image of the as-grown graphene films on Cu; (**b**) high-magnification FESEM image of a bilayer graphene sheet on a Cu grid, prepared by hot filament thermal CVD (reprinted with permission from [193]).

In another study, wrinkles within the grown graphene film can be identified using FESEM, Hawaldar et al. used FESEM to observe graphene sheet synthesized by hot filament thermal CVD (HFCVD) [193]. Wrinkles are clearly shown in the FESEM images as well as the identification of monolayer and bilayer regions within the grown graphene film was identified by the change in FESEM image contrast in Figure 27.

**Figure 28:**
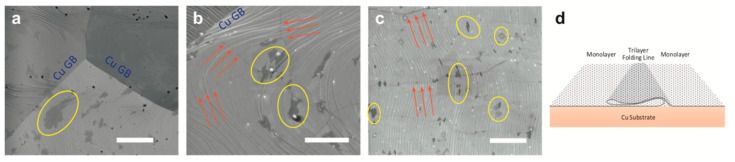
SEM images of CVD has grown graphene on Cu using different detectors (**a**) backscattering, (**b**) ETD and (**c**) TLD. Yellow circles present few-layer graphene, red arrows show the graphene folding lines and the blue Cu GB text present a Cu grain boundary. Scale bars for a–c are 5 µm; (**d**) a schematic illustration of a folding line (reprinted with permission from [138]).

The SEM technique has been demonstrated as an essential tool in identifying features within the grown graphene film such as wrinkles, defects, folding lines, number of layers and residues that are caused by the wet transfer process. Lee et al. showed how to utilize SEM to identify the aforementioned features of graphene grown by customized CVD growth [138]. They found that the SEM contrast of fold lines within graphene film is highly influenced and depends on the detector type. They showed that the SEM image by backscattering electrons of the folding lines are invisible, while they are identified using secondary electrons SEM images collected by the Everhart–Thornley detector (ETD) and through the lens detector (TLD). The brightness of the folding lines appeared lower than the ones for monolayer graphene. However, the contrast of the folding lines was better in the SEM images taken by TLD rather than in ETD. In addition, dark spots were observed and identified as regions of few-layer graphene (Figure 28).

The accelerating voltage also plays an essential rule in graphene characterisation using SEM, as typically the standard condition is using 5 kV. Higher accelerating voltages usually improve the lateral resolution and signal to noise ratio but increase the interaction volume and the penetration depth that signal from the sample’s surface is drastically decreased. Thus, it was investigated to use a lower accelerating voltage in order to increase the surface signal. The aforementioned group, Lee et al., investigated lowering the electron acceleration energy to 1 kV, and they showed that more details were observed compared to the typical SEM images taken using 5 kV. Graphene folding lines were brighter and more distinguished than monolayer graphene as well as bright spots were observed that are believed to be defects in the graphene sheet that may generate more secondary electrons [138].

**Figure 29:**
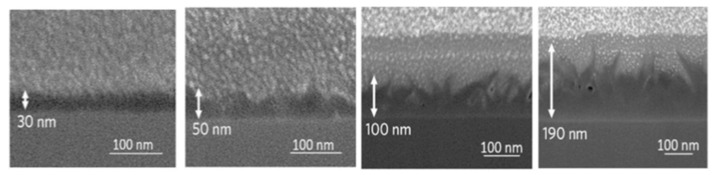
SEM images of VAGNAs at different CH_4_ ratios using PECVD, showing the variation in thickness (reprinted with permission from [101]).

Al-Hagri et al. demonstrated that SEM images helped in determining the height change, from 30 nm to 190 nm, of the vertically aligned graphene nanosheet arrays (VAGNAs) upon increasing the CH_4_ ratio using PECVD (Figure 29) [101].

In conclusion, SEM has been extensively used as a powerful tool for graphene characterisation, as numerous amounts of information can be extracted from SEM images. Optimising the SEM operating conditions such as acceleration energy, working distance and determining the right detector enables efficient visualisation of surface features in the grown graphene via CVD methods.

### 4.4. Atomic Force Microscopy

Atomic force microscopy (AFM) is an extensively used microscopic characterisation technique for graphene characterisation. 3D images obtained using AFM enables the measurement of the thickness of graphene films and the lateral dimensions, which, in turn, allows for accurately determining the number of layers present, thermal conductivity, electric and optical properties of graphene-based products [194,195].

The necessary component of the AFM instrument is a nanometre-sharp tip created using micro-fabricating technology, which is attached to an adjustable cantilever. The cantilever serves as the transducer to sense the sharp tip on a graphene sample, while a tip acts as a probe that scans over a sample line-by-line [163]. The interaction between the sharp tip and the material surface generates a force on the cantilever. The origin of this force lies in the van der Waals interaction, in which a couple of uncharged atoms in close proximity exert a force on each other. The force value is used to determine the surface height of the studied sample [196]. 

The initial step in setting up the AFM device for spectroscopic and imaging analysis involves mounting the probe in the cantilever, which is typically done with forceps. The removal of the probe for storage is done with vacuum tweezers. Correct mounting is a critical requirement for accurate measurements and ideal laser alignment [197]. The interpretation of the signals generated by the interactions between the sharp tip and the sample surface enables the determination of numerous parameters. The significant advantages of the AFM are extremely high vertical and lateral force resolutions and the availability of a variety of modes that allow probing different mechanical, physical, electrical, magnetic, frictional and elastic properties of graphene, and even to investigate nanotribological phenomena [198].

The determination of the number of layers is the most common application of the AFM. The procedure typically involves scanning the edges of graphene sheets at the places where the stacked layers form steps [199]. In most cases, the thickness of graphene flakes on substrates is measured using the AFM in tapping mode. However, the correlation between actual thickness and measured thickness, as well as and the identification of the exact number of layers are challenging. Various studies reported a wide range of measured values for the thickness of single-layer graphene, from 0.4 to 1.7 nm [194]. The measurement depends on several factors, including the choice of the substrate, relative humidity, adsorbed moisture, electrostatic interactions between the graphene surface and the sharp tip and incorrect choice of AFM parameters (e.g., free amplitude value) [200,201]. To accurately determine the exact number of graphene layers, a combination of the AFM technique and Raman spectroscopy or Tip Enhanced Raman Spectroscopy is needed [195,196].

To date, several AFM-based electrical characterisation techniques are available, a detailed review of which is given in a study by Hussain et al. [202]. Contact Mode, also known as Conducting-AFM (C-AFM), is the most commonly used AFM tool for electrical measurements. The single-layer graphene exhibits isolating behaviour along the direction perpendicular to the layers. This phenomenon fundamentally distinguishes this material from graphite that is characterised by high conducting properties due to the presence of delocalized electrons between the layers [194]. Table 2 lists the various AFM-based electrical characterisation techniques, their signal of detection, and their respective measured parameters.

The AFM in nanoindentation mode serves as an effective means of testing the mechanical properties of graphene materials. The procedure involves measuring the intensity of subtle interactions between the sample surface and the probe during the loading stage and the unloading stage and recording them on a force–distance curve. The analysis of such curves enables the determination of numerous characteristics and mechanical properties of graphene-based thin-films, including the hardness, stiffness and Young’s modulus [197,201]. The friction force microscopy (FFM) AFM mode is often used to measure the friction coefficient under various loads. The topography measurement mode can estimate the wear immediately after the end of the friction test [198].

**Figure 30:**
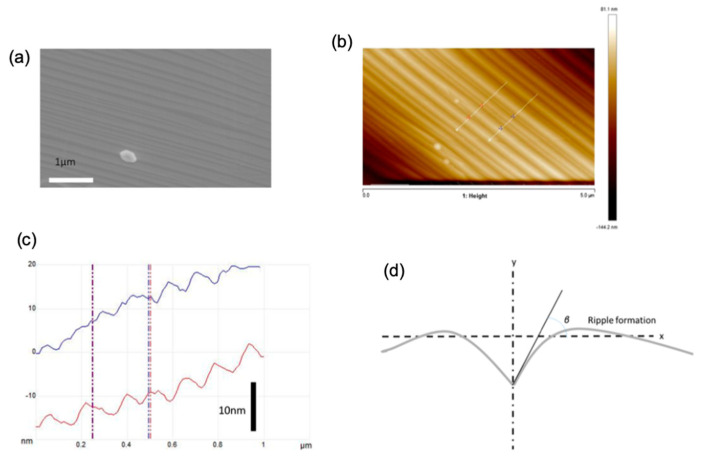
(**a**) SEM image showing the ripples in the graphene surface (4 ripples/1 µm); (**b**) AFM image of the formation of ripples in graphene; (**c**) height profile of the two sections shown in (**b**); (**d**) section showing a profile of an ideal thermal groove (reprinted with permission from [78]).

Other morphological features in graphene film can be characterised using AFM such as graphene ripples. Chaitoglou and Bertran have studied the effect of growth temperature on CVD grown graphene where ripples were observed on the surface. AFM was utilized to obtain hight measurements for the ripples; the temperature showed no significant effect on the geometry of the ripples. However, the measured step height gave a value of 5 nm, which is in agreement with the calculated depth value using the model of Mullins (Figure 30) [78]. 

**Figure 31:**
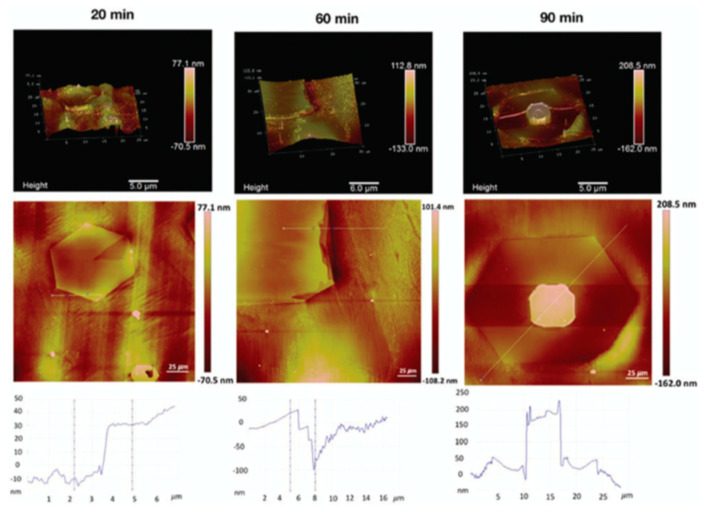
AFM images showing surface topography with a quantitative height line scan across hexagonal growth defects on graphene films grown on Cu/Mo substrate using APCVD (reprinted with permission from [49]).

In a previous article, AFM was utilized to study the nature of hexagonal domains on the surface of graphene film grown using APCVD on liquid Cu substrate and using Mo as the crucible material; these hexagons were absent in the case of using W as the crucible material. These observed hexagons showed no graphene characteristic peaks; therefore, AFM was used to investigate whether they are holes within the graphene film or precipitates on top of the graphene surface. AFM height measurement revealed that those hexagons sit on top of the graphene films, and with further elemental and thermodynamic analysis, the nature of the hexagons was identified as Mo_2_C. The thickness of the Mo_2_C hexagonal domains increased with growth time, giving heights of approximately 40, 140 and 190 nm for growth times 20, 60 and 90 min, respectively (Figure 31) [49].

**Figure 32:**
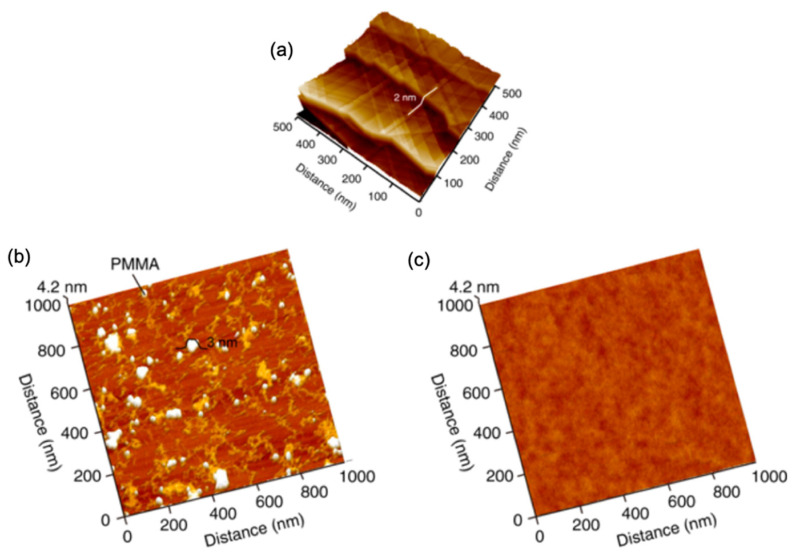
AFM images of (**a**) super-clean graphene freshly grown on Cu substrate, and transferred graphene on target substrate (**b**) unclean graphene and (**c**) super clean graphene (reprinted with permission from [203]).

Lin et al. addressed the issue of the contamination on graphene’s surface after the CVD process and demonstrated a design of Cu substrate architecture that leads to super-clean graphene film [203]. They have used a designed substrate that consists of Cu foil and Cu foam which showed remarkable results as demonstrated by AFM topographic images (Figure 32). Interestingly, the wet transfer method of graphene from its growth substrate to target substrate such as SiO_2_/Si usually result in a considerable amount of PMMA residue that profoundly affects the electronic properties of the transferred graphene film. However, it was shown that transferring super-clean graphene ensures an apparent reduction of impurities introduced from the transfer process. AFM was used to evaluate the cleanliness of the graphene surface before and after the transfer.

### 4.5. Transmission Electron Microscopy (TEM)

Transmission electron microscopy (TEM) is a powerful spectroscopy technique that provides valuable information about the structure of a material at the atomic level [204]. It was first discovered by Ruska and Knoll in 1931, and includes a wide range of analytical tools, imaging techniques and contrast mechanisms [205]. Scanning transmission electron microscopy (STEM) and high-resolution transmission electron microscopy (HR-TEM) are two widely employed TEM modifications for CVD graphene characterisation that provide versatile and rich information at superior spatial resolutions. These microscopy techniques are often used in combination with energy-dispersive X-ray analysis (EDX) or electron energy loss spectroscopy (EELS) to gain a deeper understanding of the functional and compositional characteristics of material samples [206].

**Figure 33:**
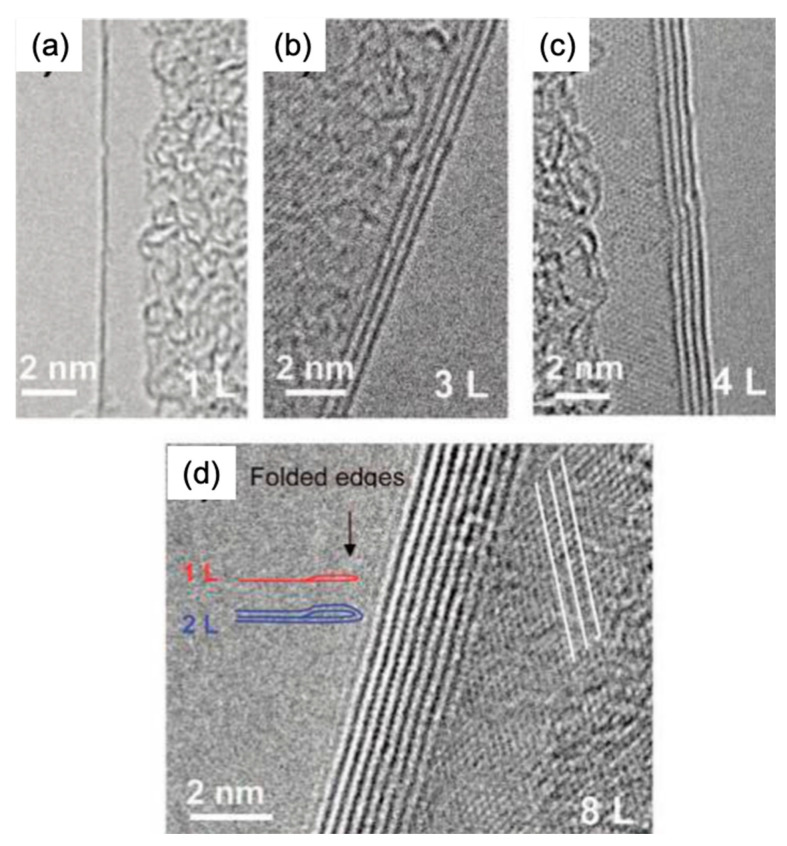
TEM images of CVD grown graphene showing different thicknesses, (**a**) 1 layer, (**b**) 3 layers, (**c**) 4 layers and (**d**) 8 layers. (reprinted with permission from [43]).

The TEM is one of the most versatile and reliable methods for studying graphene edges. The low atomic number of graphene implies decreased scattering of the electron beam, which, in turn, allows for sharper TEM images. STEM and HR-TEM imaging are practical tools for the following objectives: determining the number of layers, studying the atomic structure and characterising the electronic properties of both graphene and graphene doped with heteroatoms [207]. In addition, TEM techniques can detect 3D corrugation and ripples, recognize lattice mismatch and orientation, provide a rapid mapping of vast areas for grain boundaries and resolve atomic-scale and stacking sequence defects [208]. In a typical TEM image, the edge of each graphene layer is represented by a dark line. Counting the number of these dark lines allows researchers to accurately determine the number of layers in the synthesized CVD graphene material. Figure 33 shows the HR-TEM imaging of single-layer, tri-layer, four-layer, and eight-layer graphene sheets at the folded edge [43].

**Figure 34:**
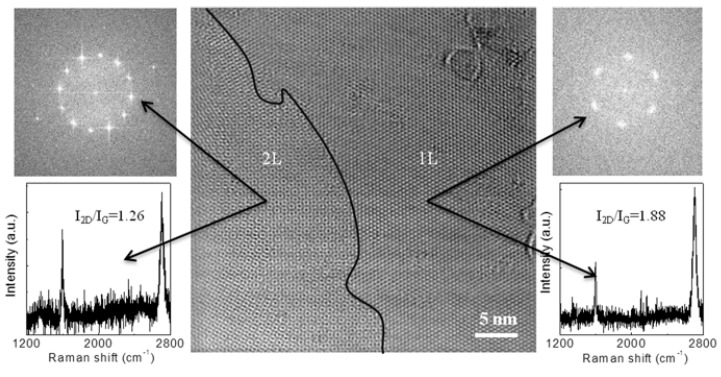
TEM analysis of CVD graphene flake from ethanol prepared with 4 h growth time. The HRTEM image showing both monolayer and bilayer graphene regions Moire patterns as a result of rotational misalignment of the two graphene layers are observed in the bilayer region. Hexagonal selected area electron diffraction patterns of the monolayer and bilayer graphene reveal the superior crystallinity. Their representative Raman spectra are illustrated in the bottom panel with *I_2D_*/*I_G_* ratio of 1.88 and 1.26 for 1 L and 2 L, respectively (reprinted with permission from [209]).

**Figure 35:**
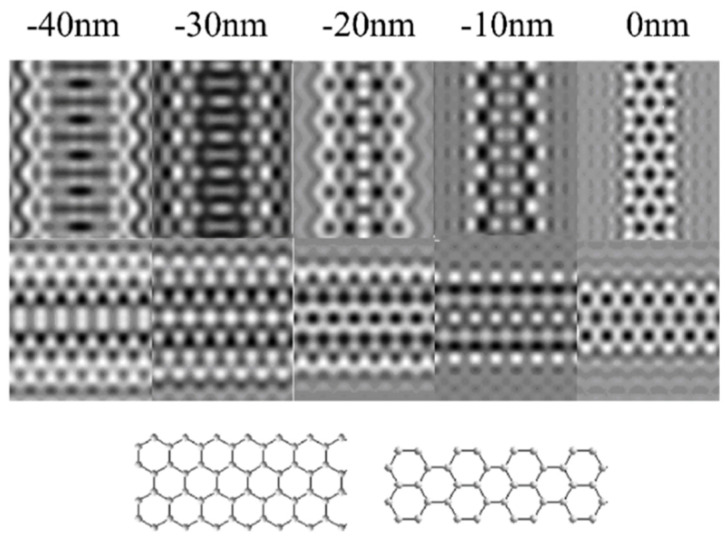
Computer simulation of HRTEM contrast showing zigzag and armchair ribbons of monolayer graphene layers (reprinted with permission from [210]).

TEM is also used to rotational misalignment between graphene layers using Moire patterns while Fourier transform of the electron diffraction pattern provides information on the nature of the graphene film as demonstrated by Nayak (Figure 34) [209]. In addition, Plachinda et al. demonstrated how low-voltage, aberration-corrected, HRTEM provides an effective tool for the investigation of graphene film structure showing the difference of armchair and zigzag ribbons of monolayer graphene layers (Figure 35) [210]. 

TEM is also considered as an excellent tool to study the structural defects in graphene which have a strong influence on the properties of the produced film that will limit their implementation. Point defects cause undesired electron scattering that drastically reduces electrical conductivity. Bonds around defects become weaker, which decrease the mechanical strength and affect the thermal conductivity. Therefore, understanding of the formation and the nature of those defects in graphene structure is essential in order to tailor the growth conditions and substrate properties to get high purity graphene with no defects. A review on the structural point and line defects in graphene were reported by Banhart et al. as they demonstrated the theoretical defect formation (intrinsic and extrinsic) as well as the utilisation of TEM as an imaging technique for those defects [211]. Defects in CVD grown graphene that can be imaged using HRTEM and STEM vary from point defects (Stone-Wales defect) [212]; in this type of defect, a total of four hexagons are transformed into two pentagons and two heptagons by rotating carbon-carbon bonds by 90°, it does not involve any addition or removal of an atom. Another example of defects in graphene is single vacancies where the graphene (or any material) is missing a lattice atom and multiple vacancies which may consist of two single vacancies or the removal of two neighbouring carbon atoms. Carbon adatoms in the perfect graphene layer cause a hybridization change so it will not be sp^2^ only, but sp^3^ hybridization might appear locally.

**Figure 36:**
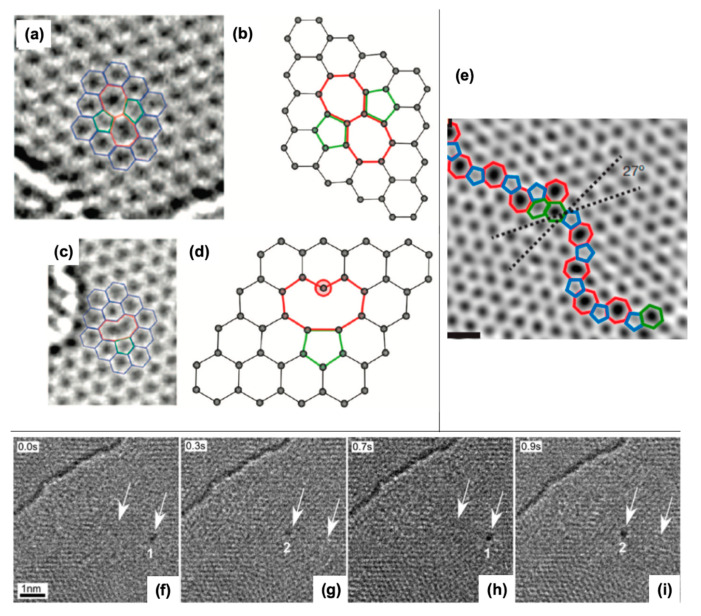
(**a**,**b**) illustrate the TEM a defect formed by rotating a carbon-carbon bond by 90° and its atomic structure as obtained from DFT calculations, respectively [211]. (**c**,**d**) show a single vacancy by experimental TEM and its atomic structure obtained by DFT calculation, respectively. (**e**) aberration-corrected annular dark-field STEM of line defects in graphene [216]; (**f**–**i**) jumping of foreign W atom (arrowed) on the surface of few-layer graphene eat 480 °C using in situ HRTEM, the repeating jumping between the trapping sites (1 and 2) at a distance of 1.5 nm shows the attraction between the metal adatom and the defect (reprinted with permission from [211]).

Similarly, adatoms defects can occur by a foreign atom (non-carbon) which affect the graphene properties; the effect will primarily depend on the interaction bond between the foreign adatom and the nearest carbon atoms [213]. Substitutional impurities are defects that lattice carbon atom is replaced with a foreign atom. In some cases, it can be induced intentionally in the graphene sheet in order to alter its electronic properties such as when using boron or nitrogen atoms as they move the position of Fermi level, changing graphene’s band gap [214,215]. In addition, line defects when graphene is grown by CVD may grow in different directions influenced by the surface morphology of the growth substrate that eventually leads to different graphene orientations along the substrate [173]. A summary of some graphene defects that can be imaged at the atomic level using TEM is presented in Figure 36.

Graphene can also be considered an ideal TEM support film. CVD graphene provides supports of extreme physical stability, periodic structure, single-atom thickness and ballistic electrical conductivity [206]. The ultrathin graphene can be used to obtain conventional and atomic-resolution TEM images of organic surface molecules, DNA, protein and the interfaces between soft and hard nanomaterials (e.g., Au nanoparticles) at a level that surpasses any other available support film [217].

## 5. Applications of CVD Graphene 

Due to its unique set of material properties, including exceptional carrier mobility (~200,000 cm^2^/Vs), outstanding flexibility, high transparency in near-infrared and visible light, graphene and graphene-based materials can be applied in a wide variety of applications in electronics, energy, biomedicine and environmental engineering [160,218,219]. The list of graphene application fields includes energy conversion and storage (solar cells, fuel cells batteries and super-capacitors), biosensors, antimicrobial material, field-effect transistors, sensing, drug delivery, tissue engineering, sorbents and membranes, etc.) [199,200]. The outstanding carrier mobility makes graphene grown by CVD an ideally suited material for several specific applications in the electronics industry, including touch panels, logic circuits, bioinspired devices, sensing materials for tactile sensors and semiconducting channels for field-effect transistors [22,24,81]. Graphene also serves as a promising alternative to indium tin oxide traditionally used as a transparent conductive material in optoelectronic devices like OLEDs [220]. 

### 5.1. Electronics Applications

The properties presented by graphene make it ideal for electronics and could even be a substitute for silicon in electronic circuits. Noting that the zero bandgap in graphene makes it challenging to be integrated in systems require 0–1 (on–off), hence, intensive research is ongoing in order to induce a bandgap in graphene to boost its use digital electronics applications. However, the range of applications for graphene in electronics extends far beyond just digital systems. Here, we briefly review the implementation of graphene in light emitting diodes (LEDs) and nano electromechanical systems (NEMS), highlighting how this helps to develop novel sensing devices and wearable electronics for health monitoring. 

#### 5.1.1. Light Emitting Diodes (LEDs)

Since the invention in 1962, LEDs have been used almost universally in a considerable number of different electronic devices. Generally, LED voltage is relatively high because it is required to be equal to or higher than the bandgap energy. These semiconductor light sources necessitate electrodes with high electrical conductivity and high transmittance to enhance their optical and electrical characteristics. However, transmittance and conductivity are mutually contradictory properties, which leads to significant difficulties for simultaneously satisfying both requirements [221]. A set of unique material properties, including high optical transmittance, excellent electric and thermal conductivity, environmental stability and mechanical flexibility, makes graphene a promising candidate for a variety of applications in optoelectronic devices. Graphene and graphene-based materials can be employed as various components of light-emitting diodes (LEDs), such as emitting layers, interfacial layers and electrodes [222]. Graphene has already found a commercial application in standard LED light bulbs as an additional graphene coating for LED filament. The first generation of such graphene LED lighting has been available on the market since 2015. In the case of standard LED light bulbs, elevated temperatures are a common cause of premature failure and significant intensity loss over the lifetime of a device. The graphene coating helps to dissipate heat, with the result that the energy efficiency of such LED light bulbs is at least 10% higher than that of standard LED light bulbs. This means that the same brightness can be achieved with less wattage.

Today, indium tin oxide (ITO) remains the most widely utilized transparent conductive electrode (TCE) in optoelectronic devices. However, the scarcity of indium inevitably leads to a constant increase in the cost of ITO, severely limiting its use for low-cost, sustainable and transparent thin-film devices [223]. The synthesis of large-area graphene films by the CVD method has significantly accelerated the application of graphene as a TCE in various optoelectronic devices. In order to become a replacement for commercially used TCEs, the material is required to possess the following properties: optical transmittance of more than 90% in the visible region and a sheet resistance below 600–800 Ω sq^−1^. Although the transmittance of graphene (about 90%) satisfies the requirement, the sheet resistance of CVD-grown graphene exceeds the acceptable level for industrial applications. Several doping methods that employ metal hybrid composites have been proposed to improve the electrical and structural properties of graphene-based materials [223].

A study by Chae et al. describes a novel electric-field-induced doping treatment, which can effectively reduce the sheet resistance of CVD-grown monolayer graphene while retaining its high transmittance. The treatment involves the injection of metallic ions (Ni^2+^) as dopants from the upper electrode into monolayer graphene films grown by the CVD method through conducting paths formed in aluminium nitride (a sacrificial buffer layer) under the application of an electric field. The authors reported the studied material retained a very high optical transmittance of about 95% after the doping process, while the work function of graphene increased from 4.36 eV to 5.0 eV as a result of p-type doping effects of Ni atoms. The LED with the Ni-doped monolayer graphene demonstrated superior light output power characteristics compared to both LEDs with a 100-nm-thick ITO electrode and LED with undoped graphene without any significant losses in the current–voltage characteristics [223].

The percolated metal nanowire networks satisfy practically all of the requirements of an excellent TCE. The low price of Cu (about 1/100 of the price of ITO) and industrial mass synthesis makes this metal an especially attractive candidate for use as TCE in optoelectronic devices. However, the application of Cu nanowires in LEDs is severely limited by the low resistance contact and poor stability against oxidation. Huang et al. proposed a CVD method for fast gas-phase encapsulation of the graphene shell layer on Cu nanowire networks to effectively improve the optoelectronic performances of TCEs and enhance oxidation resistance. The authors reported the successful fabrication of fully transparent GaN-based LED chips on a wafer using the Cu graphene nanowire TCEs. The central emission peak of this device is located at 437 nm in the blue region without any additional peaks associated with impurities, which indicates the excellent light transmission and high stability [221].

One of the promising applications of graphene-based material in the field of LED devices is their use as a replacement for conventional sapphire substrates for growing semiconductor materials for LEDs. A study by Li et al. reports the successful growth of an InGaN/GaN blue LED on wet-transferred multilayer graphene. The use of graphene as a substrate allows for releasing the stress during the growth of indium gallium nitride, which leads to a noticeable decrease in the number of cracks. The authors reported that the blue InGaN/GaN multiple quantum wells LEDs grown on graphene exhibit a higher output power and a slightly reduced crystalline quality of GaN compared to that of the LEDs grown on a conventional sapphire substrate [224].

Despite the outstanding set of desirable material properties, the implementation in stretchable electronic applications has been severely inhibited by the tendency of graphene-based materials to crack at small strains [225]. Defects present in graphene grown by CVD have a detrimental effect on both mechanical properties and other impressive physical properties. Unwelcome abrupt failures of defective graphene due to stress corrosion cracking in environmental conditions have been reported. Three existing methods for obtaining stretchable graphene are graphene origami, kirigami and the formation of composites by combining graphene with either polymers or 1D conductive species [226].

#### 5.1.2. Nano Electromechanical Systems (NEMS)

Nano electromechanical systems (NEMS) are devices that integrate electrical and mechanical functionality. Graphene provides a high surface to volume ratio, which leads to high sensitivity in the NEMS while maintaining a stable mechanical properties. Moreover, graphene’s geometry makes it suitable for standard lithography while its stiff yet flexible material and its electrical conductivity enable its integration in electrical transducers [227]. In another words, transduction, which is the transfer of mechanical motion into another form, for example, electrical signal, is expected to be highly sensitive in graphene. Crucial factors that must be in the integrated material in NEMS in order to evaluate the system’s performance are resonant quality (RQ) factor and frequency reproducibility where high value for both is desirable to obtain a high sensitive sensor or signal processor. For instance, resonators using single crystal silicon, which is considered as a thinner type of Si-based resonators often have a RQ that does not exceed 1000 nm^−1^ [228]. On the other hand, Barton et al. measured the RQ in monolayer CVD grown graphene resonators, giving a value of 14,000 nm^−1^ at room temperature [229]. Their reported results demonstrated that graphene-based resonator which are considered very thin, showed an exceptionally high QR compared to other mechanical resonators. Miao et al. provided an experimental demonstration that QR in graphene-based resonator is inversely proportional to temperature, which shed the light on understanding the dissipation mechanism in ultra-thin membrane resonators [230]. Graphene resonators in NEMs have been implemented in exciting applications as sensors for mass, force and position. Muruganathan et al. studied monolayer CVD grown graphene resonator for mass sensing of light weight gas molecules (hydrogen and argon) under varying pressure levels [231]. The sensor used graphene nanoribbons of 1 µm length and 500 nm width that were obtained by patterning the monolayer graphene on Si/SiO_2_ substrate using lithographic techniques, and metal contacts were made of Cr/Au (5/100 nm). The resonant frequency and QR were measured in vacuum condition in a chamber, followed by a set of measurements while a mixture of hydrogen and argon were injected, thus changing the pressure in the chamber. The results showed the change in adsorbed molecules (mass in zeptogram) with the pressure change which proves the efficiency of graphene NEMS-resonators for spectrometry applications for light mass molecules. Another interesting implementation of graphene based NEMS-resonator is the research conducted by Andelic et al. for the detection of chemical warfare agents (CWAs) where they applied mathematical methods and frequency shift method to detect an attached CWA molecule [232]. Fan et al. developed a robust route for realizing membranes based on bilayer CVD grown graphene and suspending large silicon proof masses on membranes with high yield that is compatible with a wafer-scale industry for MEMS/NEMS [233]. 

Another interesting property in graphene is that it exhibits an excellent piezoresistive effect, which makes it ideal for piezoresistive pressure and strain sensors. In 2013, Smith et al. developed a highly sensitive pressure sensor using monolayer CVD grown graphene membranes that were suspended over the patterns on SiO_2_ [234]. The sensitivity of the piezoresistive graphene sensor surpassed the one obtained using silicon and CNTS-based pressure sensors. Fan et al. reported the use of CVD grown graphene in NEMS piezoresistive transducers to fabricate ultra small spring–mass systems using suspending silicon proof masses on bilayer graphene ribbons [235]. The results showed that the obtained transducers have the potential to be used for accelerometers in NEMS where a drastic reduction in size is possible compared to a state-of-the-art conventional silicon based accelerometer. Such ultra-small accelerometer can be extremely useful in wearable devices to monitor activity and patient recovery process as well as implantable nano-devices to monitor heart failure. However, a challenging issue associated with integrating sensors on flexible substrates for flexible electronics and wearable electronics is that the sensing process and sensitivity are temperature dependent. Nag et al. performed a set of experiments comparing a graphene piezoresistive material and a polysilicon by applying pressure at varying temperatures to analyse the sensor degradation behaviour at different temperatures [236]. Both sensors showed similarly close sensitivity at low temperature, but graphene based sensor showed higher sensitivity at elevated temperatures, indicating the possibility of utilizing graphene pressure sensors at high temperature. Humidity effect on the sensing performance was studied by Kim et al. as a transparent and flexible self-activated all-graphene-based sensor was fabricated for NO_2_ sensing [237]. They presented the linearity between the NO_2_ concentration and the sensor response, while the performance of the graphene-based sensor was evaluated in dry and humid conditions (0% and 50% humid environment) showing a less than 5% degradation in humidity, which is much lower than those of metal oxide gas sensors under the same conditions. Some excellent dedicated reviews about graphene in NEMS have been reported, discussing both NEMS and MEMS applications [238,239,240].

#### 5.1.3. Biosensors and Flexible Wearable Devices

As aforementioned, it was demonstrated that the implementation of CVD grown graphene in conventional NEMS structures where they still consist of transistor-like nanoelectronics shows superior performance and sensitivity [241]. Traditional NEMS usually use silicon-based substrates (silicon, polysilicon and amorphous silicon), which are rigid, expensive, low biocompatibility and require complex processes; therefore, alternative material for flexible devices are highly desirable to utilize the remarkable graphene properties and performance in NEMS in flexible electronics such as flexible displays and wearable biosensors [242]. An effective health monitoring has been always an interesting area for research in order to provide better healthcare and increase the quality of life. Hence, the integration of biosensors that employ different sensing techniques with high sensitivity responses is surely an area for intensive investigation. Briefly, a biosensor is an analytical device that has the ability to detect biomolecular elements using an suitable transducer to generate a measurable signal [243]. Meanwhile, biosensing covers a wider range of sensing capabilities including heart rate, blood oxygen level, body motion, cancer cells, etc. A typical biosensor system consists of a bioreceptor that must has the ability to sensitively recognize biomolecules of interest such as certain enzymes, RNA, DNA, viruses, etc. This bioreceptor is interfaced with a transducer to generate the measurable signal. Typically, graphene and graphene-derivatives have been demonstrated as ultra-sensitive material for precise biosensing. Among the different graphene derivatives (Go, rGO) and the synthesis techniques, CVD grown graphene gave the best sensing performance due to the higher quality and low/negligible defects density since defects lead to serious degradation in graphene’s properties [226]. 

Chen et al. reported the implementation of PECVD grown graphene nano walls (GNWs) in the fabrication of flexible electromechanical biosensor for real-time measurement of lactate [244]. In the method, crosslinking was adopted to functionalize the obtained GNWs with chitosanglutaraldehyde to immobilize lactate enzyme on GNWs using PET as the flexible substrate. Next, the conductive electrodes using carbon ink were printed on the graphene/PET and the reference electrodes were also printed using AG/AgCl conductive ink. Finally, an insulator made from dielectric paste giving a cost-effective method to fabricate biosensor with reported results that reflect high sensitivity and stability. Wang et al. demonstrated a simple and low cost method to fabricate a strain sensor for human motion detection using graphene woven fabrics (GWFs) grown by PECVD [245]. The flexible sensor was attachable to a human body to sense motion since it was flexibly adhered on PDMS and medical tape composite. A strain was applied causing cracks in the structure that subsequently decreased the current path ways and increased the resistance. This was applied to detect various human motions such as bending finger, elbow, wrist and knee showing the difference in the obtained sensing signals when walking and running. The graphene-based strain sensor responded well to weak human motion representing high sensitivity. Another life-saving application is the non-invasive approach for glucose monitoring for Type 1 and Type 2 diabetes. Lipani et al. proposed a path-selective, transdermal glucose monitoring system by using pixels of array consisting of CVD graphene and enzyme-encasing hydrogel reservoir that detect the glucose level through selective hair follicles pathways across the skin [246]. The glucose sensor using graphene film decorated with Pt nanoparticles to increase the detection sensitivity while the whole system is supported by a flexible substrate. The careful monitoring of the sensing performance tracking blood sugar provided a proof of concept of the proposed method as the sensor worked efficiently for 6 h. Interestingly, the described approach ensures that the measurements reflect actual blood glucose with high precision without being subjected to movement or individual skin characteristics like other wearable devices.

### 5.2. Energy Applications

A combination of superior mechanical strength, flexibility, high optical transparency and superb carrier mobility makes graphene a great candidate to be used in energy applications. Here, we discuss three examples of using CVD grown graphene in this category, briefly reviewing the utilisation of graphene in solar cells, batteries and supercapacitors. 

#### 5.2.1. Solar Cells

Solar energy is the most abundant sustainable form of energy on the planet. Earth receives 3 × 10^24^ J of solar radiation per year, which is approximately 10,000 times the current global energy consumption. Thus, as solar energy has great potential in providing energy security, research is continuously tackling the main challenges in solar cells and photovoltaics (PVs) in order to reduce the cost and find novel approaches to increase their efficiency. The current market of conventional solar cells is dominated by crystalline silicon which is a well-established technology. Recent research reported Si-based solar cell with an efficiency of 26%, which is close to the theoretical limit of 29.1% [247]. However, the high cost remains the main drawback. Another interesting category that holds much potential is the thin film PVs that have various types such as CdTe [248], CIGS [249], perovskite [250] and CZTS [251] showing that recent thin-film PVs have reached efficiency as high as a 20% conversion rate [252]. Researchers working on the development of PVs have raised a great interest in implementing graphene, owing to its remarkable properties, the potential of further reducing the cost while increasing efficiency. Considering the high electron mobility, transparency, conductivity and flexibility as well as physical and chemical stability, graphene has been studied as the ideal material in solar cells whether as a contact or interface layer in mostly all types of PVs (Si, thin film, organic, etc.) [253]. However, despite the exceptional properties in graphene, there are still challenges regarding its efficiency. Li et al. conducted the earliest studies in implementing graphene in solar cells using a graphene/silicon Schottky junction and had reported an efficiency of 1.5% [254].

Since then, many studies have been made in order to increase efficiency by using an interfacial layer, doping and antireflective coating. Song et al. in 2015 reported a drastic increase in efficiency after using an optimal oxide interfacial layer, followed by a further increase to reach 12.4% after chemical doping followed by 15.6% efficiency by applying an antireflective coating, demonstrating a method for the combination of the three aforementioned techniques [255]. Later, Li et al. have used CVD grown graphene to fabricate graphene/GaAs van der Waals heterostructure solar cell with an efficiency of 18.5% by designing a graphene-dielectric-graphene gating structure [256]. Their method consists of growing monolayer graphene on Cu substrate using hot-wall CVD under the flow of a mixture of hydrogen and methane, followed by reducing the native oxide layer from GaAs using HCl (10 wt.%, 3 min). Au was thermally evaporated on the backside of GaAs with a thickness of 60 nm while the insulating layer under the graphene/metal contact was made of 80 nm of SiN_x_ dielectric layer which was deposited using PECVD with a designed lithography mask on the front surface of GaAS. The exposed area in the dielectric layer is considered as the active area for the graphene/GaAs solar cell, where the CVD graphene was transferred to the active GaAs area. The performance of the fabricated solar cell was tested using a solar simulator giving 18.5% power conversion efficiency (PCE) while drift–diffusion simulation was utilised and higher PCE was predicted with over 23.8% shedding the light to great potential of utilising graphene in PVs by investigating more dielectric materials [256]. Furthermore, recently, AbdulRehman et al. reported the use of PECVD to grow graphene at low temperature directly on silicon substrate to be used as a Schottky junction solar cell eliminating the need for graphene transfer process [257]. Graphene as zero band gap material works as metal and silicon as the semiconductor, creating a Schottky junction. The effect of the grown graphene thickness was studied showing that 4 nm is the optimum thickness to be used in graphene/silicon Schottky junction with an efficiency of 5.51%. Further treatment using chemical doping (HNO_3_) and antireflecting coating of PMMA were performed on the optimum graphene thickness in order to increase the PCE, reaching an efficiency of 9.18% [257]. 

Another type is the Perovskite solar cells, especially combining crystalline silicon in the design of the solar cell, is predicted to have high PCE using a hybrid of perovskite/silicon tandem devices. However, such a design requires the use of transparent conductive electrodes. Conventional transparent conductive oxides cannot be directly deposited on the top surface of the perovskite cell, requiring buffer layers leading to current losses. Promising alternatives are electrodes made of the silver nanowire (AgNW) as they have low sheet resistance, giving an efficiency of 17%; however, their optical transmission is 87%, which is far below that of graphene [258]. Lang et al. reported that the use of CVD had grown graphene as the transparent contact (conductive transparent electrode) to increase the efficiency in tandem solar cells [259]. The results showed an excellent electrical performance using graphene contact, similar to the typically used gold contacts; however, the obtained optical transmittance was impressively high and a PCE of 13.2% which is higher than perovskite single junction by 30%.

Organic solar cells (OSCs) have gained a worldwide interest due to their potential of having flexible and cost-effective solar cells. Organic solar cells consist of substrate, anode, hole transport layer, absorber, electron transport layer and a cathode. The anode layer is typically a transparent conductive layer made of indium tin oxide (ITO) for its electrical conductivity and optical transparency. However, ITO is brittle, making it unsuitable for the next generation of flexible devices. Additionally, since indium is a strategic material, alternatives are very desirable for the industry. Graphene has been proposed as the ideal replacement for ITO due to its properties. The efficiency of flexible OSCs is still low compared to other types of solar cells. However, efforts are ongoing to develop their efficiency further. Kim et al. investigated the use of CVD grown graphene as the transparent conductive electrode on flexible PET substrates [260]. They showed an optimal graphene thickness of three layers; taking into consideration the sheet resistance and transmittance optimisation based on the number of layers, a PCE of 4.33% was achieved. Interestingly, a more recent study by Koo et al. showed the direct integration of polyimide (PI) to functionalised CVD grown graphene as it played a dual role, as a carrier film for graphene and as a substrate for the graphene electrode [261]. The PCE of 15.2% and optical transmittance of 92% were obtained using the functionalised PI/graphene flexible OSCs, which surpassed most of the reported efficiencies in this category.

#### 5.2.2. Batteries

Batteries are usually categorised as rechargeable, which mainly consists of lithium and non-rechargeable such as alkaline and zinc-based batteries. Briefly, the non-rechargeable contain two electrodes and an electrolyte where the charges flow in one direction and usually have a short lifespan. While rechargeable, it also consists of two electrodes, where the cathode is made of lithium-based metal oxide, and the anode is a porous carbon and having lithium ions flow in both directions (charging/discharging). The increasing demand of mobile electronic devices (e.g., laptops, cell phones) requires high energy and power density storage systems that provide fast charging and stable performance. The traditional lithium-metal oxide batteries have many limitations, making them unable to meet those requirements [262]. A suggested solution was the use of lithium-sulfur (Li-S) batteries due to their high energy density, natural abundance of sulfur material and low cost [263]. Despite the aforementioned advantages, Li-S exhibit serious drawbacks: short lifespan, low efficiency and fast capacity decay rate due to poor kinetics of sulfur electrochemistry as well as the high-dependency of sulfur on the temperature and they suffer from poor high-temperature performance [264]. These drawbacks are mainly originated from the low conductivity of elemental sulfur and the fact that the produced polysulfides (Li_2_S_x_, x = 4, 6 and 8) are highly soluble that they dissolve and shuttle through the electrolytes [265] and between the Li anode and the S cathode. Therefore, many attempts have been made to overcome those issues including the enhancement of electrical conductivity of produced S-species, suppressing the polysulfides shuttling by trapping polysulfides in cathode materials (e.g., graphene, CNTs, carbon spheres) by functional separator as well as modification of electrolyte [266].

Therefore, the need for a conductive material that is thin with negligible weight and yet flexible to be used as separator coating, CVD grown graphene film arise as the most promising candidate to enhance the power and energy density of Li-S batteries. Du et al. prepared a Li-S battery with polypropylene (PP) separator that is coated with two layers of CVD grown graphene [267]. Enhanced performance of Li-S battery was reported in their work owing, as the reason behind this advancement, to the fact that the intactness of the conductive CVD graphene layers on the PP separator led to minimising the diffusion of soluble polysulfides and converting it into insoluble Li_2_S_2_/Li_2_S, which suppressed the shuttle effect. Therefore, this decreased the self-discharge compared to a conventional Li-S battery using a standard PP separator without sacrificing energy density. In another study, Assegie et al. reported an anode-free Li-metal battery consisting of a Cu current collector that is modified and coated with multilayer CVD grown graphene [268]. They found that the graphene on Cu worked as an artificial solid electrolyte interface (SEI) that stabilises electrode interface, encapsulating deposited lithium and block lithium-dendrite (a dendrite begins when lithium ions start to clump to form a needle-like growth “dendrite” on the surface of the lithium metal “anode”). This suppressing effect of dendrite formation reduces the undesired reactions that drastically reduce the energy density of the battery.

More studies showed the potential of using nanoscale silicon in anodes for lithium-ion batteries since they have high cycle stability and reversible capacity compared to bulk silicon [269]. The size reduction in nano-silicon effectively improves the charge-cycle performance due to its role in promoting lithium-ion diffusion, shortening the path between the silicon nanoparticles and the electrons in the electrolyte while reducing the transmission time. However, it still cannot withstand long-term charge-discharge stability. Thus, more stable material is required to form a nano-silicon composite. Zhang et al. have effectively fabricated a nanocomposite consists of silicon nanoparticles, and CVD has grown graphene on Cu substrate [270]. Their design that started with spin-coating Cu substrate with dispersion consists of Si nanoparticles (Si-NPs), followed by CVD graphene growth process leading to the formation of Si-NPs/graphene nanocomposite film. A second design used CNTs dispersion as an auxiliary dispersing agent, testing different concentrations of CNTs and Si-NPs to get the optimal CNTs/Si-NPs/graphene nanocomposite film to be used as the anode material in the lithium-ion battery. It was shown that CNTs effectively avoided Si-NPs agglomeration in the dispersion followed by spin-coating on Cu and then CVD graphene growth. The nanocomposite was tested as an anode in button lithium-ion battery giving a high energy capacity, yet it still suffered from a short life cycle, suggesting further studies on the stability of Si-NPs/graphene nanocomposite. Saulnier et al. also studied the use of multilayer CVD grown graphene on nickel substrate as the anode material [271]. The effect of films’ morphological defects on the sustainability of discharge capacity at high speed was highlighted. They showed the essential role of edge plane that usually exist in multilayer graphene, in facilitating lithium-ion diffusion. Therefore, it was concluded that defects’ engineering in CVD grown graphene could be an interesting area for further research to enhance the quality performance of graphene-based lithium-ion batteries.

#### 5.2.3. Supercapacitors

Supercapacitors have attracted enormous interest in research communities due to their outstanding potential in terms of high power density, high charge/discharge rates and long lifespan performance. They are mainly divided into two categories based on the mechanism of their energy storage; (1) electric double-layer capacitors (EDLCs) and pseudo-capacitors [272]. The EDLCs use carbon electrodes, and the energy storage occurs at the interface between the conductive electrode and the electrolyte. In contrast, the pseudo-capacitors electrodes made of metal oxide or conductive polymer and the energy storage occur due to Faradaic electron charges transfer between electrode and electrolyte through redox reactions. Therefore, in order to achieve high storage and electrochemical performances in both types as well as ensuring stable life cycles, enhancing electrical conductivity is a necessity. Thus, due to graphene physical, chemical and electrical properties, the interest arises to utilise it in high energy storage supercapacitors. In monolayer graphene, the theoretical calculated specific capacitance is ~550 F g^−1^ if the entire surface area is utilised [273,274], but the practical performance of graphene capacitance still falls behind its theoretical potential due to process-induced impurities and agglomeration. Among the different synthesis methods used to produce graphene, CVD grown graphene is the most promising form in supercapacitors due to its uniformity, large crystalline nature and low defects density. 

Li et al. were the first to report the capacitance of graphene/tantalum (Ta) electrodes [275]. They synthesised a hot-filament CVD grown multilayer graphene on tantalum (Ta) wires with a thin buffer layer of TaC using several CVD conditions. The supercapacitor was assembled by using the graphene/Ta wire as the anode and platinum (Pt) wire as the cathode immersed in 1 M of Na_2_So_4_ electrolyte, while Ag/AgCl was used as a reference anode. The supercapacitor performance showed an excellent charge/discharge cycling stability, and it sustained 84% of its initial capacitance after 1000 cycles. This revealed that graphene/Ta wire electrode is such a promising electrode material for high energy storage devices since it exhibited the highest capacitance of 345.5 F g^−1^. A recent study on reducing the equivalent series resistance (ESR) was reported by Kwon using optimised CVD grown graphene on Ni foil using different cooling rates (20, 40 and 100 °C/min) [276]. Graphene was used as an interface contact layer between the current collector (Ni foil) and the electrode material (reduced graphene oxide rGO). They demonstrated that faster cooling rate led to a reduced ESR showing that supercapacitor fabricated using CVD graphene (cooling rate = 100 °C/min) gave the best capacitance retention of ~99% after 3000 cycles at high scan rate (100 Vs^−1^). This was explained by the reduced ESR, thus providing a better interface, enabling more contact points between the electrode and the current collector. This shows advancement of the implementation of G-based electrodes in a supercapacitor for devices that require faster scan rates.

Another interesting implementation of 2D grown graphene films via CVD is their ability to be used in flexible solid-state (the electrolyte is non-liquid) supercapacitors. All-solid-state supercapacitors attracted the most attention due to their simple design, flexibility and efficiency in energy storage, enabling their use in wearable devices [277]. Early studies in flexible all-solid-state supercapacitors have used fibre-based materials (e.g., carbon and non-carbon based fibres); however, the overall capacitance remained poor in the range of 1.7–41 mF/cm^2^. This was followed by more efforts indirectly use self-assembled and chemically modified graphene sheet to fabricate graphene-based fibres employing facile and spinning processes [278,279]. Li et al. reported the development of a novel approach to directly create graphene fibres from graphene grown films using CVD [280]. The novel yet simple approach used to convert the 2D CVD grown graphene film into a 1D graphene fibre-like structure using an organic solvent followed by drying to give the porous structure with produced fibres thickness of 20–50 µm followed by decorating the structure with low wt.% of MnO_2_. The results showed remarkable porous structure (uniform pore distribution) and electrochemical performances in terms of discharge capacitance and cycling stability shedding light on its potential use as an electrode in supercapacitors. Later, the same research group (Li et al.), studied the implementation of their previously reported graphene fibre structure derived from CVD grown graphene into a flexible all-solid-state supercapacitor using gel electrolyte made [281]. The graphene-based (graphene fibre + MnO_2_ nanoparticles) structure worked as the active electrode and electron collectors. The areal capacitance of the graphene-based supercapacitor was reported to be 42.02 mF cm^−2^, which was considerably high compared to other fibre-based supercapacitors. In addition, the structure withstood mechanical bending and exhibited stable galvanic cycling as well as electrochemical performance.

More recent study was reported by Kim et al. in 2017, a novel roll-based patterning technology for CVD grown graphene was presented using highly durable Ni stamps to pattern the graphene at room temperature [282]. The method is mask-less and photoresist-free technology, as it starts with growing graphene using CVD on Cu substrate, followed by patterning the graphene using a roll-to-plate transfer machine and finally etching the Cu foil and transfer the patterned graphene onto a target substrate. A transparent supercapacitor was then fabricated using the patterned graphene as the electrode and electron collector, and the gel electrolyte (PVA/H_2_SO_4_) dropped cast to the supercapacitor design. The reported area specific capacitance of 5.0 µF cm^−2^ using the fabricated micro-supercapacitor was comparable to other reported supercapacitors based on CVD has grown graphene [283,284].

## 6. Conclusions

An in-depth review was conducted on the synthesis of graphene using CVD methods (hot-wall, cold-wall and plasma enhanced) and the main characterisation techniques to study the characteristics of the produced graphene (Raman, SEM, AFM and TEM). A detailed review on the used growth substrates including the morphological effect and the nucleation and growth mechanism in solid and liquid substrates. Finally, an overview on graphene integration in applications, highlighting electronics and energy sectors in particular.

In this review, the various factors affecting the quality of graphene film and its growth process were discussed, including the system total pressure, partial pressure of hydrogen and hydrocarbon species, growth temperature, source of power, etc. Knowledge of the effect of growth conditions and their correlation with the physical and chemical properties of the growth substrate is essential in creating a fundamental understanding of the process. In addition, it gives a powerful tool for tuning the growth parameters accordingly to achieve the desired graphene film, whether, monolayer, bilayer, few layer, or doped graphene which directly influence the electronic properties of the resulted graphene film. The progress in synthesis techniques and exploring wider range of parameters space in order to gain control over the deposition process to have the ability for large scale production while maintaining high quality graphene properties. Growth substrates were discussed in terms of the effect of pre-treatment methods and the surface morphology on the uniformity of the grown graphene film. Liquid substrates show the most potential to grow uniform, single crystalline monolayer graphene with no or a negligible amount of defects due to the absence of grain boundaries and other morphological defects when the growth starts on liquid state substrate. However, more investigations need to be done to explore other substrates such as low-melting point metals and precisely monitor the shrinkage effect of the substrate on the grown graphene.

Overview of the essential characterisation techniques were discussed. Utilising Raman spectroscopy, scanning electron microscopy, atomic force microscopy and transmission electron microscopy reveals a tremendous amount of information about the characteristic properties of the produced graphene film as well as the defects nature and density. In addition, progress in the integration of CVD grown graphene in electronics and energy applications was discussed. Graphene’s role in the impressive performance enhancement of LEDs, NEMS, biosensors, solar cells, batteries and supercapacitors were highlighted. The window for graphene applications is wide open for limitless fields. However, CVD methods produce the best electronic quality for graphene, which makes it ideal for electronics and energy technologies in particular.

Future prospects shall include novel designs for cost effective CVD systems that allow the continuous growth of high quality graphene on a large scale. Moreover, synthesising functionalized graphene and other 2D materials need to be researched as it could pave the way for a wider range of possibilities. Such novel materials can be employed and be very beneficial in enhancing the performance of electrochemical and electromechanical micro and nano devices. For example, their integration in ultra-sensitive biosensing for healthcare and medical sector as well as environmental sector (toxins detection, anti erosion and anti corrosion coatings) and evaluating the performance under harsh conditions are interesting fields that require further studies.

## Figures and Tables

**Table 1:** Summary of reported literature of graphene growth conditions using different types of CVD methods.

CVD Method	Pressure (mbar)	Reaction Gas	Flow Rate (sccm)	Temperature (°C)	Growth Time (min)	Substrate	No. of Graphene Layers	Ref.
	800	Ar:H_2_	-	600, 800, 900	~250	Ge/Si wafer	ML	[121]
**Hot-Wall**	750	CH_4_/(Ar/10%H_2_)	-	1050	30	Cu	ML, multilayer	[153]
	Atmospheric	n-C_6_H_14_	1	1070	60	Ni	3–4 layers	[154]
	26.67	H_2_/CH_4_	400/200	1000	5	Cu	ML and FL	[155]
	13.33	H_2_:CH_4_	6:50	1000	4	Cu	multilayer	[139]
	133.3	C_2_H_4_:Ar	0.1–0.2 vol% 30–60	975	1.5	Cu	monolayer	[140]
	53.32	H_2_:CH_4_	10:0.1	1030	30	Cu	bilayer	[51]
	1013	CH_4_	100 ppm	1060	60	Cu	monolayer	[156]
	13.33	H_2_:CH_4_	200:10	1020	20	Cu	multilayer	[146]
	1013	Ar:H_2_:CH_4_ H_2_:CH_4_	800:10:0.5 300:22	1080	4–6	Cu	Monolayer-dendritic G-flakes Monolayer-Circular G-flakes	[157]
	1013	Ar:H_2_:CH_4_ H_2_:CH_4_	(300–600):(20–5):(5–20) 300:(6–10)	950–1030 1080–1120	15–120 5–30	In, Ga Cu	Monolayer Monolayer	[149]
	1013	Ar:H2:CH_4_	300:(0–30):(5–20)	1020	10–60	Ga	Monolayer	[50]
	1013	H_2_:CH_4_	300:7	1100	30	Cu	Monolayer	[148]
	1013	H_2_:CH_4_	500:(3–5)	1100,1025	380	Pt_3_Si/Pt	Bilayer	[152]
	1013	Ar:H_2_:CH_4_	500:200:1.5	1070, 1090	25, 5	Cu	multilayer	[150]
	1013	H_2_:CH_4_	300:6	1120	28 min–4 h	Cu on W	Monolayer	[50]
	1013	Ar:CH_4_:H_2_	300:(5–20):(0–30)	1120	5–120	Ga, In, Cu	Monolayer	[149]
	1013	Ar:CH_4_:H_2_	200:(0.15–5):(0–100)	1090	15, 45 and 90	Cu on W or Mo	Monolayer	[158]
	1013	Ar:CH_4_:H2	200:(0.15–5):(0–100)	700–1100	0.5–30	Ga. In, Sn, In-Cu, Sn-Ni and Sn-Ag-Cu	-	[159]
	1013	Ar:CH_4_:H_2_	200:10:80	1090	90	Cu on W or Mo	Monolayer	[63]
		H_2_:CH_4_	120:6	1120	5–90	Cu on W and Mo	Monolayer	[49]
**Cold Wall**	27	Ar:N_2_ H_2_:CH_4_	100:700 100:8	1000 100/min	30	Ni	Monolayer	[87]
	27	H_2_:CH_4_	80:1	450–750	30	SiO_2_/Si	Monolayer	[91]
	0.0133	H_2_:CH_4_	0.4:1.4	1000	20	Cu	Monolayer	[81]
	1	H_2_:C_2_H_2_	24:12	550–1000	30	-	6–8 layers	[160]
	666.612	H_2_:CH_4_	3 to 400:13	1030	50	SiO2/Si	Mono-Bi Layer	[161]
	15	H_2_:CH_4_	60:15	750	20	SiO_2_/Si	Monolayer	[80]
	27	H_2_:CH_4_	4:1	900–1050	20	SiO_2_/Si	Monolayer	[82]
	144	H_2_:CH_4_	8:1	900–1000	30	Cu	Monolayer	[22]
	1013	H_2_:CH_4_	1500:25	900–1000	10	SiO_2_/Si	2 to 3 layers	[162]
	11	H_2_:CH_4_ Ar	10:23 120	900–1000	33	PET	Monolayer	[83]
	0.1	H_2_:CH_4:_Ar	20:60 140	800–1060 300/min	45	SiO_2_/Si	Bilayer	[84]
	0.02	H_2_:CH_4:_Ar	-	1000	30	SiO_2_/Si	Multilayer	[86]
	7	H_2_:CH_4:_Ar	50:0.25:500	900–2000	30	Cu	Monolayer	[89]
	93	H_2_:CH_4:_Ar	0.7:280:10000	360–1000	30	Cu	Monolayer	[163]
**MW-PECVD**	500 mTorr	Ne:H_2_: CH_4_	-:2–5-	<420	5–20 min	Cu	Monolayer	[164]
	8–13 mbar	Ar:H_2_:CH_4_	100:50:1–10	700–850	30–90 s	Co	Multilayer	[98]
	1 × 10^−6^ mbar	H_2_:C_2_H_2_:NH_3_	240–360: 20–40:0–120	700–750	-	Al_2_O_3_, SiO_2_	Monolayer Multilayer	[96]
	10–25 mbar	Ar: H_2_: CH_4_	-:50:1–10	700–1000	30–90 s	Co-Cu	Multilayer	[165]
	30 mbar	H_2_:CH_4_	130–150:50–70	900	30–45 min	Si(100), SiO_2_	Multilayer	[166]
	25 mTorr	Ar: H_2_: CH_4_: O_2_	H_2_: 2.5 (mono), 2 (bi)	400 (bi), 425 (mono)	5–15 min	Cu	Mono, Bi Layer	[97]
**ECR-PECVD**	5.4 × 10^−2^ mbar	H_2_:C_2_H_2_	55:0.25–0.20	650	300 mint–10 h	quartz and silica	Monolayer	[99]
	10^−6^ Torr	H_2_:CH_4_	200:20–180	-	5 min	Cu	GNWs	[167]
	10^−6^ mbar	H_2_:C_2_H_2_	50:0.8–0.24	550–650	150 min	Si/SiO_x_	Mono, multi-Layer	[168]
**RF-PECVD**	200 mTorr–5 Torr	Ar:H_2_:CH_4_	20:20:3	400–700	20 min	Glass, Si/SiO_2_ wafers, Cu	Multilayer	[104]
	450 mTorr	Ar:CH_4_	100:2	700–750	5–30 min	SiO_2_/Si	Multilayer	[132]
	<10^−2^ mbar	H_2_:CH_4_	2:10	600	15 min	Cu	Multilayer	[169]
	570mTorr–1.2 Torr	Ar:H_2_:CH_4_	50:10:0.1	750–1000	30–120 min	quartz	Multilayer	[170]
	400 mTorr	Ar: H_2_:CH_4_	400:60:40	625	90 min	Ge	Monolayer	[102]
	-	Ar:H_2_:CH_4_	10:20:7	750	30 min	Cu	Mono, multi-Layer	[171]
	4.1 × 0^−3^ Torr	CH_4_	0.2–1	800	10–30 min	Si plane, textured Si wafers	Multilayer	[103]
**HF-PECVD**	-	H_2_:CH_4_:	-	1600	5 min	Ni	Multilayer	[105]
	6 × 10^−4^ Pa	N_2_:H_2_:C_2_H_2_	0–20:50:1	850	9 mint	glass	Multilayer	[106]
**ICP-PECVD**	191–209 mTorr	Ar:H_2_:CH_4_	21.5:10:2–4	300	10 s	Cu/SiO_2_/Si	Bilayer	[108]
	1 Torr	Ar:H_2_:CH_4_	100:100:1	700–1000	5 s−60 min	Cu, Fe_2_O_3_ film	Mono, multi-Layer	[107]
	0.8–1.5 Pa	Ar:C_3_H_8_ Ar:N_2_:C_3_H _8_Ar:SiH_4_:C_3_H_8_	C_3_H_8_:50–400	350–500	1–30 min	Cu	Multilayer	[109]
	470–875 mTorr	Ar: H_2_:CH_4_	50:10–50:5	850	5–300 s	Cu	Monolayer	[111]
	-	Ar:H_2_:CH_4_	180:5:5	850	1 min	Cu	Monolayer	[112]

**Table 2:** AFM techniques and their applications (reprinted with permission from [202]).

AFM Modes	Signal of Detection	Measurement of the Physical Quantity
Conductive Atomic Force Microscopy (C-AFM)	Current	Dielectric breakdown, local conductivity, doping distribution and defects
Scanning Kelvin Probe Microscopy (SKPM)	Electric potential	The work function, voltage drop, surface potential and charge transfer
Conventional Electrostatic Force Microscopy (EFM)	Electrostatic forces	Electrostatic gradient, dielectric response, and surface potential
Multi-Harmonic Electrostatic Force Microscopy (MH-EFM)	Electrostatic forces	Surface potential, mobile charge carriers and work function
Scanning Microwave Impedance Microscopy (sMIM)	Microwave reflection	Dielectric constant, charge carrier variations, permittivity and conductivity variation, doping density
Piezoresponse Force Microscopy (PFM)	Electromechanical coupling	Ferroelectric coercive field, electromechanical response, switching behaviour
